# Neural extracellular matrix regulates visual sensory motor integration

**DOI:** 10.1016/j.isci.2024.108846

**Published:** 2024-01-09

**Authors:** Jacqueline Reinhard, Cornelius Mueller-Buehl, Susanne Wiemann, Lars Roll, Veronika Luft, Hamed Shabani, Daniel L. Rathbun, Lin Gan, Chao-Chung Kuo, Julia Franzen, Stephanie C. Joachim, Andreas Faissner

**Affiliations:** 1 Department of Cell Morphology and Molecular Neurobiology, Faculty of Biology and Biotechnology, Ruhr University Bochum, 44780 Bochum, Germany; 2 Institute for Ophthalmic Research, Centre for Ophthalmology, Eberhard-Karls-University Tuebingen, 72076 Tuebingen, Germany; 3 Interdisciplinary Centre for Clinical Research Aachen, RWTH Aachen University, 52074 Aachen, Germany; 4 Experimental Eye Research Institute, University Eye Hospital, Ruhr University Bochum, 44892 Bochum, Germany

**Keywords:** Neuroscience, Sensory neuroscience, Omics, Transcriptomics

## Abstract

Visual processing depends on sensitive and balanced synaptic neurotransmission. Extracellular matrix proteins in the environment of cells are key modulators in synaptogenesis and synaptic plasticity. In the present study, we provide evidence that the combined loss of the four extracellular matrix components, brevican, neurocan, tenascin-C, and tenascin-R, in quadruple knockout mice leads to severe retinal dysfunction and diminished visual motion processing *in vivo*. Remarkably, impaired visual motion processing was accompanied by a developmental loss of cholinergic direction-selective starburst amacrine cells. Additionally, we noted imbalance of inhibitory and excitatory synaptic signaling in the quadruple knockout retina. Collectively, the study offers insights into the functional importance of four key extracellular matrix proteins for retinal function, visual motion processing, and synaptic signaling.

## Highlights

• Loss of brevican, neurocan, tenascin-C, and tenascin-R leads to retinal dysfunction
• Visual motion impairment in quadruple, tenascin-C, and tenascin-R knockout mice
• Loss of direction-selective starburst amacrine cells in quadruple knockout mice
• The matrisome influences inhibitory and excitatory synaptic balance

## Graphical abstract

**Figure:**
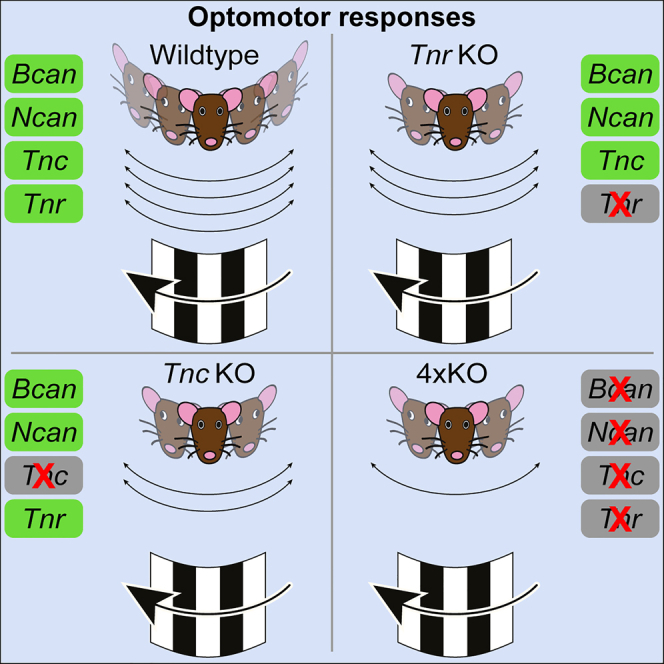

## Introduction

Growing evidence indicates that the extracellular matrix (ECM) matrisome and interacting molecules are key players in synaptic maturation and plasticity.^1^^,^^2^ The ECM is a highly organized and interactive meshwork composed of complexes of hyaluronan and proteoglycans linked by oligomeric glycoproteins.^3^ Particularly, ECM molecules of the tenascin glycoprotein family have become a research focus as important synaptic modulators.^4^^,^^5^ For example, tenascin-C (Tnc) modulates the activity of L-type Ca^2+^ channels and is expressed in specific patterns in the developing central nervous system (CNS).^6^ Thereafter, Tnc is downregulated but persists in regions of neuronal plasticity, such as the hippocampus.^7^ The tenascin family member tenascin-R (Tnr) is an important component of perineuronal nets (PNNs).^8^^,^^9^ Thus, the loss of Tnr leads to an abnormal PNN formation and an altered distribution of PNN-associated ECM molecules in *Tnr* knockout (KO) mice. Important binding partners of tenascins are chondroitin sulfate proteoglycans (CSPGs), which consist of a core protein with covalently attached glycosaminoglycan chains.^10^^,^^11^ The CNS-specific CSPGs neurocan (Ncan) and brevican (Bcan) inhibit neurite outgrowth^12^^,^^13^ and display developmentally regulated expression in the rodent CNS.^14^ Both tenascins as well as CSPGs are structural components of PNNs or closely associated with them in the brain and play a crucial role in regulating neurotransmission and synaptic plasticity.^8^^,^^15^ As such, the elimination of either *Bcan*, *Tnc*, or *Tnr* in gene KO approaches resulted in the modification of long-term potentiation (LTP) in the CA1 region.^6^^,^^16^^,^^17^^,^^18^ It is worth noting that in the retina, the formation of PNNs, as observed in different brain regions, is not as evident. In the retina, the four ECM molecules, Bcan, Ncan, Tnc, and Tnr, appear to primarily associate with the synaptic plexiform layers, although their functional significance remains unclear.^19^^,^^20^^,^^21^^,^^22^

The integration of visual motion processes relies on a precise and finely tuned synaptic neurotransmission. In the retina, starburst amacrine cells (SACs) play a pivotal role in direction selectivity and the detection of directional motion.^23^^,^^24^ SACs associate with the retinal ganglion cell layer (GCL) and inner nuclear layer (INL) and are characterized by the expression of choline acetyltransferase (ChAT). By releasing γ-aminobutyric acid (GABA), SACs modulate synaptic input on direction-selective RGCs (DSGCs).^25^ Notably, GABA_A_ receptors containing the α2 subunit have been found to be critical for direction-selective inhibition.^26^

To examine the role of the neural ECM in visual motion processing, we studied mice lacking the ECM components Bcan, Ncan, Tnc, and Tnr. These quadruple KO mice are viable and fertile.^27^ Previous investigations revealed that hippocampal neurons from quadruple KO mice exhibit diminished PNNs, altered expression of synaptic proteins, and lower miniature excitatory and inhibitory postsynaptic current (mEPSC/mIPSC) frequencies.^28^ A reduced short-term depression and changed frequency dependence was observed in the quadruple KO hippocampus *in vivo*,^29^ paralleled by a dramatic change in the ratio of excitatory and inhibitory synapses in cultured hippocampal neurons.^30^ Moreover, these ECM proteins are important regulators of the interplay between PNNs, synaptic integrity, inhibitory interneurons, and the transcription factor Otx2 (orthodenticle homeobox 2) in the visual cortex.^31^ These observations indicate that the neural ECM preserves the equilibrium of neuronal networks by stabilizing inhibitory synapses.^32^ However, it is currently poorly understood if and to which extent the quadruple KO results in an excitatory-inhibitory synaptic imbalance *in vivo*. It is also not known which functional and cellular consequences this entails in neural networks. In this perspective, we focused on retinal function in quadruple KO mice. So far, the consequences of an ECM KO for visual processing are only poorly understood.^33^^,^^34^ However, previous studies have indicated the essential role of the ECM protein pikachurin in facilitating selective synapse formation between retinal photoreceptors and ON bipolar cells, thus modulating visual motion processing and optokinetic responses.^35^^,^^36^^,^^37^

Herein, we demonstrate severe retinal dysfunction in quadruple KO mice lacking the ECM molecules Bcan, Ncan, Tnc, and Tnr. Most interestingly, altered visual motion processing was evident in the quadruple KO as well as in *Tnc* or *Tnr* single KO mice. This could be traced to a loss of cholinergic direction-selective SACs, an impaired balance of inhibitory GABAergic and excitatory glutamatergic synapses and significant changes in the transcriptome of quadruple KO retinae. Collectively, our study provides crucial evidence that the ECM influences visual sensory motor behavior already at the retina level, without intervention of disease and/or experimental manipulation.

## Results

### Differential expression of Bcan, Ncan, Tnc, and Tnr in the postnatal and adult mouse retina

**Figure 1:**
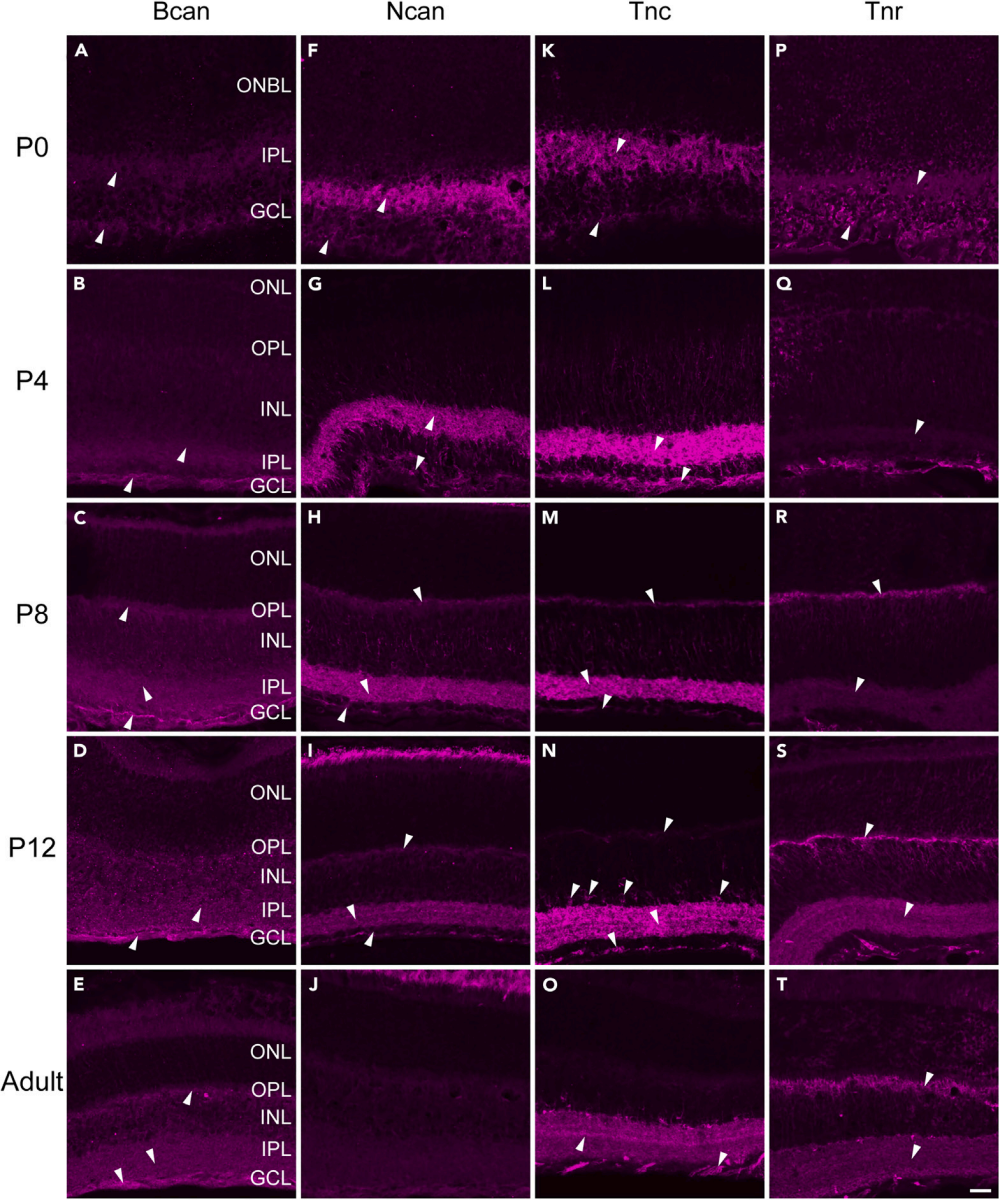
Immunohistochemical staining of Bcan, Ncan, Tnc, and Tnr in the postnatal and adult mouse retina Differential staining pattern of Bcan (A–E), Ncan (F–J), Tnc (K–O), and Tnr (P–T) in the postnatal (P0, P4, P8, and P12) and adult retina. White arrows mark prominent signals. Scale bar: 20 μm. Bcan, brevican; GCL, ganglion cell layer; INL, inner nuclear layer; IPL, inner plexiform layer; Ncan, neurocan; ONBL, outer neuroblastic layer; ONL, outer nuclear layer; OPL, outer plexiform layer; P, postnatal; Tnc, tenascin-C; Tnr, tenascin-R.

Before analyzing the functional importance of Bcan, Ncan, Tnc, and Tnr, we wanted to elucidate their detailed expression patterns during mouse retinal development via immunohistochemistry and *in situ* hybridization (Figures 1 and S1). Additionally, the specificity of the antibodies utilized for immunohistochemistry, targeting Bcan, Ncan, Tnc, and Tnr, was validated through control staining of quadruple KO retinae (Figure S2).

The expression of Bcan in the developing mouse retina has not been analyzed so far. *Bcan* mRNA expression was hardly detectable at postnatal day (P) 0 and P4 but increased throughout ongoing retinal development (Figures S1A–S1E`).

Also, on protein level, we observed weak immunoreactivity of Bcan in the inner plexiform layer (IPL) and the GCL around P0 and P4 (Figures 1A and 1B). From P8 onwards, we found signals in the outer plexiform layer (OPL), and the immunoreactivity in the GCL increased (Figures 1C–1E).

The protein pattern of Ncan was described previously in the developing rat retina.^21^ When we analyzed the mRNA expression of *Ncan* in the P0 mouse retina, we observed faint signals throughout all retinal layers (Figures S1F–S1J`). At P4, these signals became more pronounced, especially in the inner retina. However, *Ncan* mRNA expression was less distinct in the adult retina. The immunohistochemical analyses revealed Ncan protein from P0 onward in the IPL and GCL (Figures 1F–1J). At P8, Ncan was also observed in the OPL. At adulthood, no specific Ncan immunoreactivity was observed. Remarkably, Ncan signals vanished in retinal layers.

Developmental Tnc expression has been previously reported in the chick retina.^19^ Here, *Tnc* mRNA was detected in amacrine cells (ACs), displaced ACs, and horizontal cells (HCs). In our study, we revealed a similar *Tnc* expression in the developing mouse retina. *Tnc* mRNA expression was detected in single cells of the outer neuroblastic layer (ONBL) at P0 (Figures S1K and S1K`). From P4 until P12, Tnc signals were found at the basal side of the INL and in the GCL, where ACs are located (Figures S1L–S1N`). This expression pattern was also noted in the adult retina (Figures S1O and S1O`). Additionally, we observed signals at the apical side of the INL. On protein level, Tnc was localized in the IPL and GCL from P4 onward (Figures 1K–1O). At P8, additional signals were observed in the OPL. In the adult retina, Tnc immunoreactivity decreased slightly.

In the mouse retina, Tnr expression seems to increase until the third postnatal week and then remains at a constant level.^20^ Our results suggest that some cells in the ONBL already expressed *Tnr* mRNA at birth (Figures S1P and S1P`). Upon P4, *Tnr* mRNA was expressed by cells at the apical side of the INL (Figures S1Q–S1T`). Signals were also observed at the basal side of the INL and in the GCL. On protein level, only faint Tnr signals were detected at P0 and P4 (Figures 1P and 1Q). After P8, Tnr immunoreactivity highly increased in the OPL as well as in the IPL, and this expression pattern was maintained until the adult stage (Figures 1R–1T).

Taken together, our study showed that the expression of Bcan, Ncan, Tnc, and Tnr is developmentally regulated in the mouse retina. Most interestingly, on protein level, these ECM constituents showed a prominent expression in the plexiform layers, which points to their potential functional relevance at synaptic sites.

### Physiological intraocular pressure but severe reduction of a-/b-wave amplitudes in quadruple KO mice

**Figure 2:**
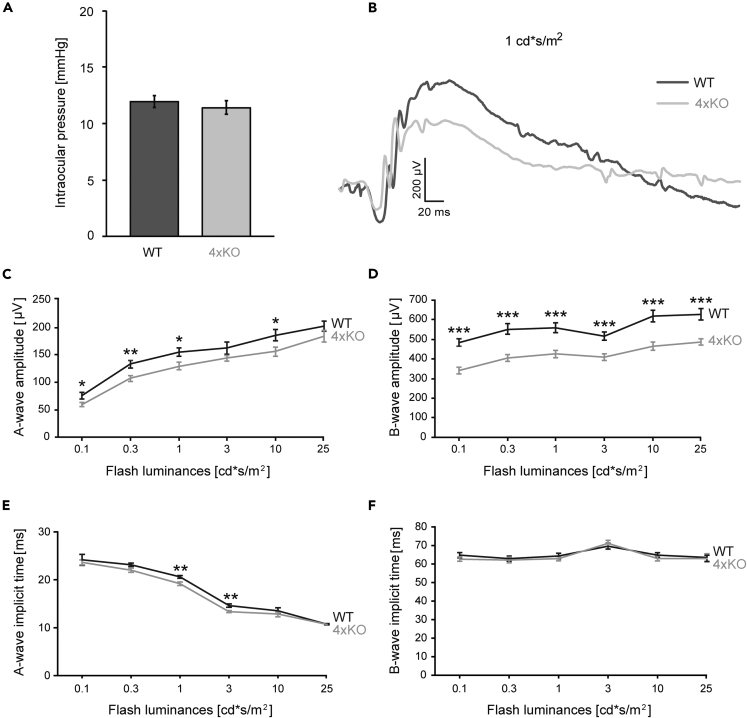
Physiological intraocular pressure but severe reduction of a-/b-wave amplitudes in quadruple KO mice (A) Physiological IOP in quadruple KO and WT mice. The IOP of both eyes was measured in quadruple KO and WT mice. Quadruple KO and WT mice exhibited a comparable, normal physiological IOP. p > 0.05; N = 12. (B) Representative ERG waveforms are shown for WT and KO eyes at a flash luminance of 1 cd∗s/m^2^. (C and D) ERG recordings revealed significantly reduced a-wave amplitudes in quadruple KO compared with WT animals. Remarkably, b-wave amplitudes were significantly decreased. (E and F) The implicit time of the a-wave was significantly reduced in quadruple KO mice at a flash luminance of 1.0 and 3.0 cd∗s/m^2^, whereas a comparable implicit time of the b-wave was noted in both groups. ∗p < 0.05, ∗∗p < 0.01, ∗∗∗p < 0.001; N = 8. 4xKO, quadruple KO; μV = microvolt; cd∗s/m2 = candela x seconds per meter square; IOP, intraocular pressure; mmHg, millimeters of mercury; ms, millisecond; WT, wild-type. Data are shown as mean ± SEM.

To explore possible physiological and functional alterations in quadruple KO mice, we measured the intraocular pressure (IOP) and analyzed the retinal function (Figures 2A–2F; Table S1). The IOP measurement was conducted to establish a potential link between ocular hypertension and the ECM gene knockout. By assessing the IOP, we aimed to investigate whether IOP changes indicate a pathological condition, such as glaucoma, and whether it might influence the outcomes of the subsequent experiments. However, tonometry analyses revealed a comparable and physiological IOP in wild-type (WT) and quadruple KO mice (quadruple KO: 11.39 ± 0.60 mmHg; WT: 11.94 ± 0.52 mmHg; p = 0.49; Figure 2A).

Functional analyses were performed by scotopic electroretinogram (ERG) recordings (Figures 2B–2F; Table S1). Here, the a-wave represents electrical responses of the rod photoreceptors, whereas the b-wave shows electrical responses of rod BCs to the light flash. ERG recordings showed that quadruple KO mice had significantly reduced a-wave amplitudes (p < 0.05 at 0.1, 0.3, 1, and 10 cd∗s/m^2^; Figures 2B and 2C; Table S1). Contrary, the implicit time of the a-wave was decreased in quadruple KO mice (p < 0.01 at 1 and 3 cd∗s/m^2^; Figure 2E; Table S1), indicating a faster photoreceptor response. Most notably, b-wave amplitudes were reduced (p < 0.001; 0.1 to 25 cd∗s/m^2^; Figures 2B and 2D; Table S1). However, a comparable implicit time of the b-wave was noted in both genotypes (p > 0.05; from 0.1 to 25 cd∗s/m^2^; Figure 2F; Table S1).

Overall, these data indicated severe retinal functional deficits in quadruple KO mice, which seemed to particularly affect the inner retina.

### Impairment of optomotor responses in quadruple KO mice as well as *Tnc* and *Tnr* single KO mice

**Figure 3:**
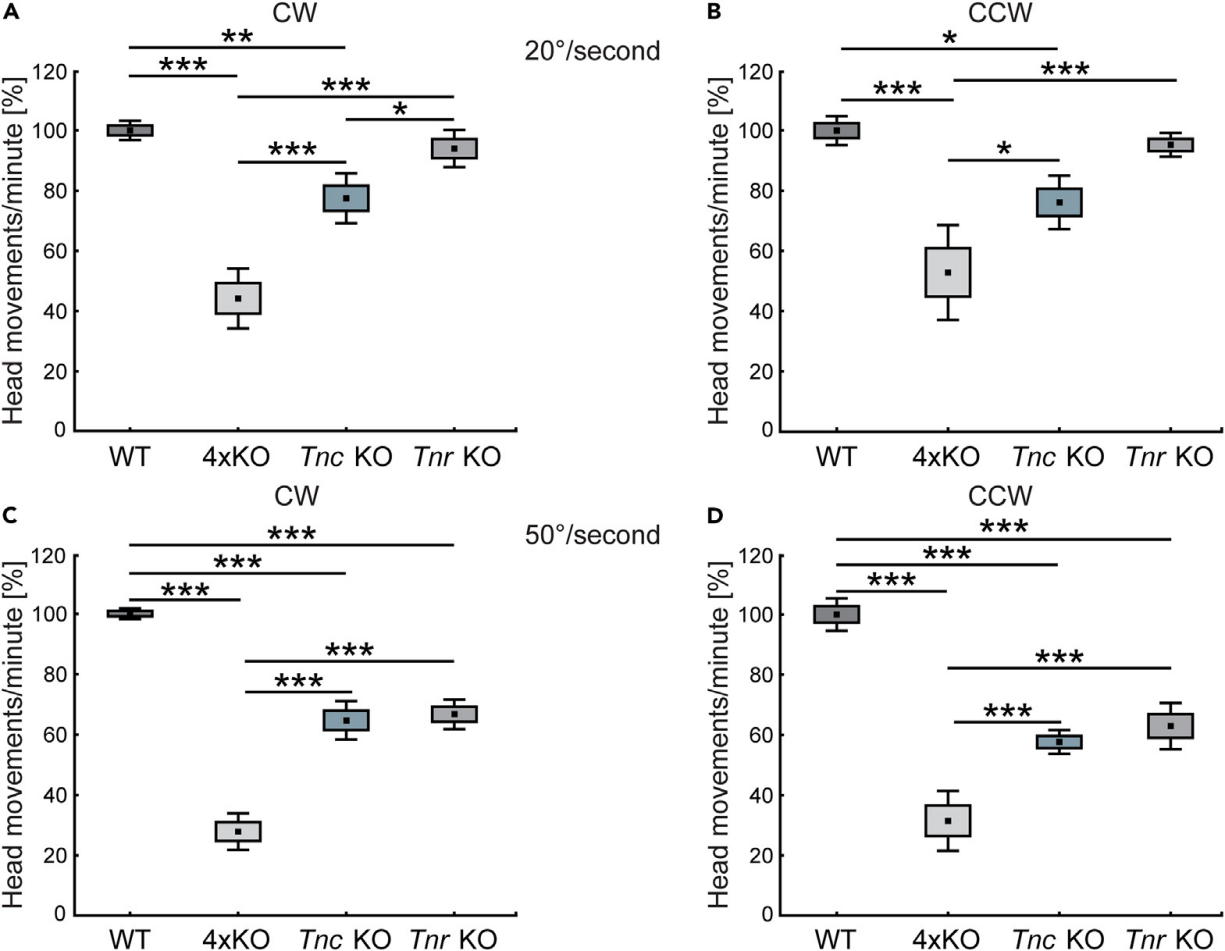
Impairment of optomotor responses in quadruple KO mice as well as *Tnc* and *Tnr* single KO mice (A–D) Quadruple KO mice showed a significantly reduced number of head movements at slow [20°/second; (A) and (B)] as well as fast velocities [50°/second; (C) and (D)] and in both directions (rotation clockwise [CW], (A) and (C); and rotation counterclockwise [CCW], (B) and (D)] compared with WT mice. Additionally, quadruple KO mice displayed a lower number of head movements in comparison to *Tnc* KO and *Tnr* KO animals. Compared with the WT group, as shown for both directions and velocities, *Tnc* KO mice exhibited a reduced number of head movements. *Tnr* KO animals displayed a comparable number of saccades at slow velocities in both directions compared with control mice. However, a significant reduction was observed at fast velocities. Only a slightly reduced or comparable number of head movements was found in *Tnc* KO when compared with *Tnr* KO mice. ∗∗p < 0.01, ∗∗∗p < 0.001; N = 6. 4xKO, quadruple KO; CW, clockwise; CCW, counterclockwise; *Tnc* KO, *Tnc* knockout; *Tnr* KO, *Tnr* knockout; WT, wild-type. Data are shown as mean ± SEM ± SD and were normalized to the WT values (mean set to 100%).

Based on our findings of an impaired retinal function in quadruple KO mice, we implemented further measurements of visual motion processing. Therefore, we measured the optomotor response (OMR), a basic mechanism to stabilize images on the retina in a moving environment.^38^ We analyzed the OMR in quadruple KO as well as in *Tnc* and *Tnr* single KO mice (Figures 3A–3D). As measure for the OMR, we counted the number of head movements. Quadruple KO mice showed a significantly reduced number of head movements/minute at slow (20°/second) as well as fast velocities (50°/second) and in both directions (rotation clockwise and counterclockwise) compared with WT mice (p < 0.001).

Quadruple KO mice also displayed a lower number of head movements in comparison to *Tnc* (p < 0.05) and *Tnr* (p < 0.001) KO animals. Compared with the WT group, as shown for both directions, *Tnc* KO mice performed less head movements at 20°/second (p < 0.05) and 50°/second (p < 0.001). *Tnr* KO animals showed a comparable number of head movements at slow velocities in both directions as WT mice (p > 0.05). However, a significant reduction was observed at fast velocities (p < 0.001). Only a slightly reduced (p < 0.05; 20°/second, clockwise) or comparable number of head movements (p > 0.05; 20°/second, CCW; 50°/second, clockwise and counterclockwise) was found in *Tnc* KO mice when compared with *Tnr* KO animals.

In summary, our data indicate a defective visual processing in the investigated ECM mutants, which was most severe in quadruple KO mice. Interestingly, compared with quadruple KO mice, *Tnc* single KO mice showed less severe impairment in the optomotor response behavior but significant limitations compared with *Tnr* KO mice. These results indicate that the four ECM molecules are critical for visual motion processing.

### Loss of cholinergic direction-selective ON-SACs in the quadruple KO retina

Because of the impairment in the optomotor behavior in quadruple KO mice, we next analyzed whether these defects are associated with deficits on the cellular level. Cholinergic direction-selective SACs play a crucial role in mediating retinal direction selectivity. Thus, destruction of SACs leads to a loss of directionality in RGCs as well as a loss of optokinetic responses, indicating their importance for stabilizing image motion.^39^^,^^40^ Additionally, selective ablation of SACs leads to a loss of OMRs.^41^

**Figure 4:**
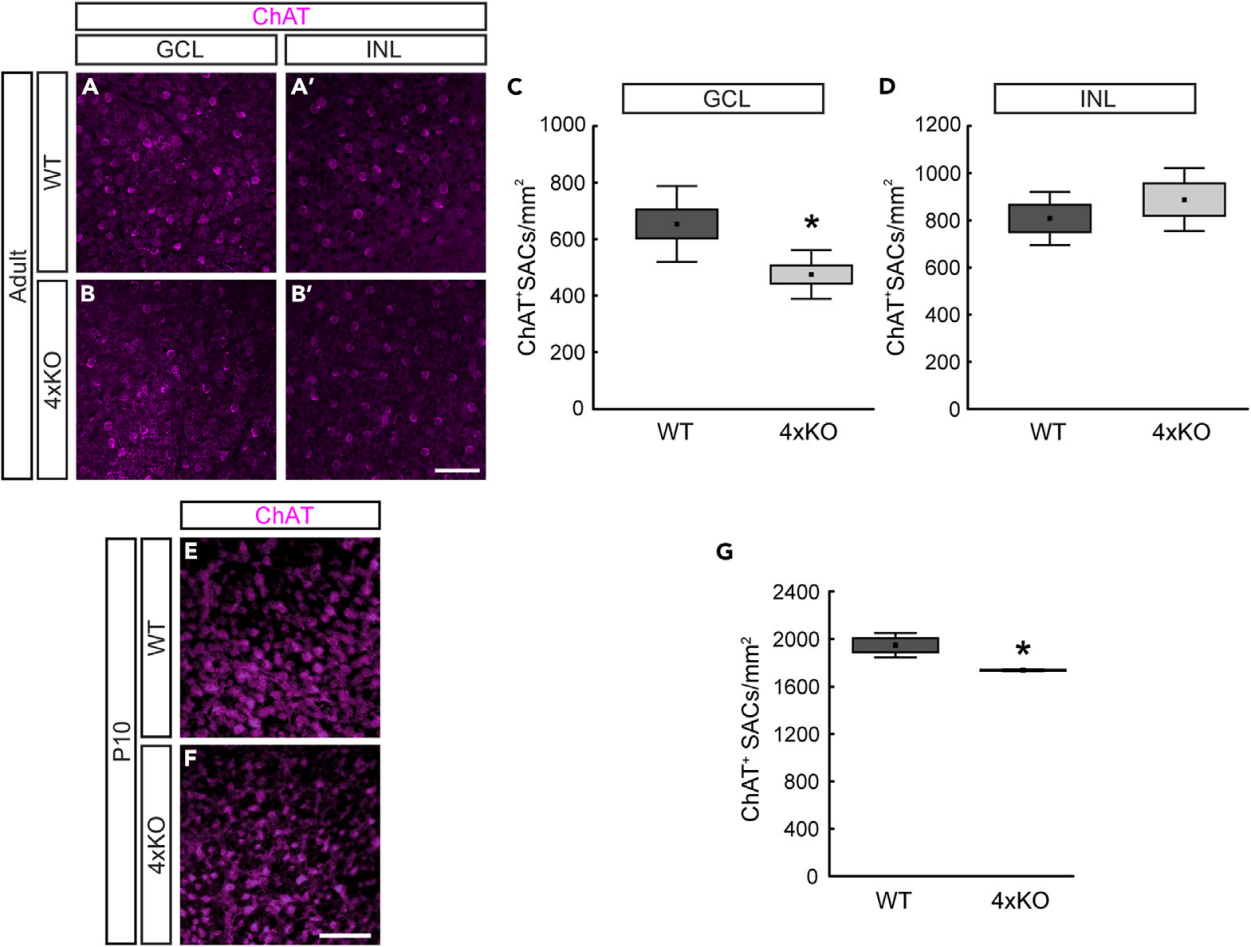
Loss of cholinergic direction-selective ON-SACs in the quadruple KO retina (A and B`) Representative photographs of ChAT^+^ SACs (magenta) in the GCL (=ON-SACs; A, B) and INL (=OFF-SACs; A`, B`) of WT and quadruple KO flat-mount retinae. (C and D) The number of ON-/OFF-ChAT^+^ cells was determined in the GCL (C) and INL (D). A significant loss in the number of ON-ChAT^+^ cells was found in quadruple KO compared with control mice (C) (p = 0.01). No alterations of OFF-ChAT^+^ cells could be noted (D) (p = 0.2). N = 7. (E and F) Exemplary photographs of ChAT^+^ SACs (magenta) in P10 flat-mount quadruple KO and WT retinae. (G) Quantification revealed a significantly reduced number of ChAT^+^ SACs in postnatal quadruple KO retinae (p < 0.05). Scale bars: 50 μm. ∗p < 0.05; N = 3. ChAT, choline acetyltransferase; GCL, ganglion cell layer; INL, inner nuclear layer; ON-SAC, ON-starburst amacrine cell; 4xKO, quadruple knockout; WT, wild-type. Data are shown as mean ± SEM ± SD.

To analyze the number of SACs in quadruple KO and WT retinae, flat-mounts were immunostained with anti-ChAT antibodies. Thus, cell bodies of ON- and OFF-cholinergic SACs were specifically stained in the GCL and INL, respectively (Figures 4A and 4B`).^42^ Quantification revealed a significantly lower number of cholinergic SACs in the GCL of quadruple KO compared with WT retinae (WT: 653.5 ± 134.6 cells/mm^2^; quadruple KO: 474.9 ± 86.0 cells/mm^2^, p = 0.01, Figure 4C). In contrast, no change in the number of ChAT^+^ cells could be observed in the INL (WT: 729.8 ± 99.7 cells/mm^2^; quadruple KO: 806.1 ± 111.0 cells/mm^2^, p = 0.2, Figure 4D). In addition, we conducted immunostainings of ChAT^+^ SACs in *Tnc* and *Tnr* single KO mice. However, we observed a similar number of SACs compared with WT mice (Figures S3A–S3D). These results indicated a specific loss of cholinergic ON-SACs in the quadruple KO retina.

In the mouse retina, acetylcholine (ACh) is required for the propagation of spontaneous activity after birth to P10.^43^ Around eye opening at P12, cholinergic neurons show a mature-like phenotype.^44^ To investigate whether the loss of ChAT^+^ SACs in quadruple KO retinae was already evident in early development, we stained retinal flat-mounts at P10 (Figures 4E and 4F). At this point in time, immature SACs are organized in the INL and GCL.^44^ However, it is challenging to assign immature SACs to the INL or GCL due to the ongoing processes of cellular migration and the incomplete differentiation and separation of the layers. Therefore, we counted the total number of SACs in both layers of the retina. We noted a significant reduction in premature total ChAT^+^ SACs from quadruple KO retinae (WT: 1949.5 ± 59.5 cells/mm^2^; quadruple KO: 1739.0 ± 4.0 cells/mm^2^, p = 0.02, Figure 4G). Based on these results, we conclude that the reduced number of SACs in quadruple KO already originates during early retinal development.

### Comparable response of DSGCs in quadruple KO and WT mice

**Figure 5:**
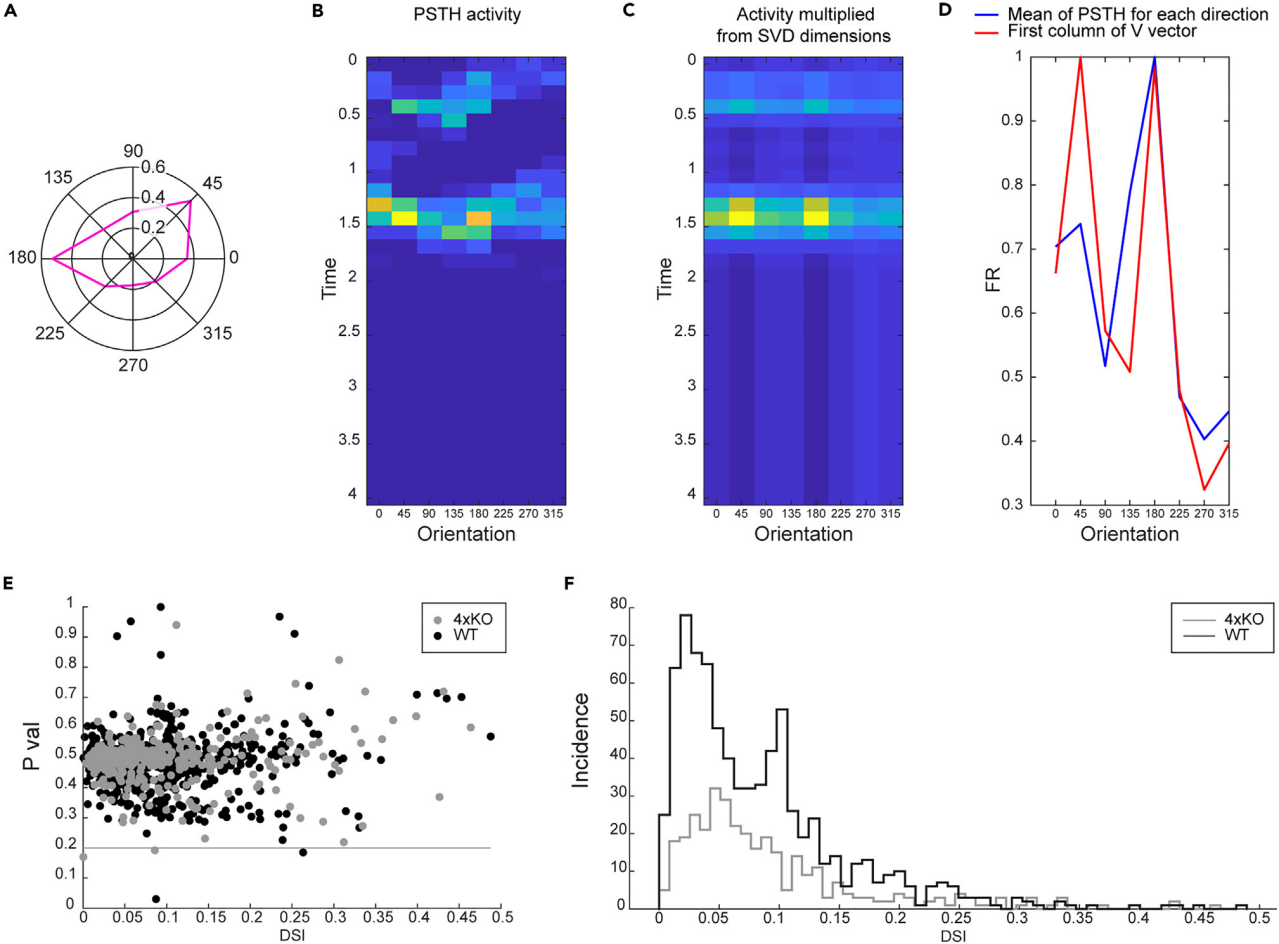
No significant change in the response of DSGCs DSGC responses were recorded in quadruple KO and WT retinae by MEA analyses. (A) The polar plot and (B) peristimulus time histogram (PSTH) show the exemplary response of an individual DSGC [magenta in (A)]. The cell mainly responded to movement of the light bar at an angle of 45° and 180° [yellow/orange in (B)]. (C) Projected data using the first column of V, drawn from SVD decomposition of the PSTH response matrix shown in (B). (D) Red: average of PSTH in (B), blue: first column of V. (E) The dot diagram displays the p value and the direction-selectivity index of WT DSGCs (black dots) and quadruple KO DSGCs (gray dots). (F) The diagram depicts the cumulative responses (incidence) of WT DSGCs (black line) and quadruple KO DSGCs (gray line). Although we observed a trend toward lower DSGC responses in quadruple KO compared with WT mice, no significant differences were found between both genotypes. N = 10. DSGC, direction-selective retinal ganglion cell; DSI, direction-selectivity index; FR, firing rate; 4xKO, quadruple knockout; WT, wild-type.

Direction selectivity of DSGCs results from patterned excitatory and inhibitory inputs during motion stimuli. A critical factor for DSGC direction-selective processing is their inhibition via SACs. Given the reduction in the number of SACs in the GCL of quadruple KO retinae, it was of great interest to analyze the electrical DSGC response to a moving object. Thus, to investigate possible changes in DSGC responses, MEA recordings, with a light bar moving in different directions as stimulus, were carried out in quadruple KO and WT retinae (Figures 5A–5F and S4). The polar plot (Figure 5A) and peristimulus time histogram (PSTH; Figure 5B) visualize the exemplary response of an individual DSGC. The cell mainly responded to a movement of the light bar at an angle of 45° and 180° (Figures 5B–5D). In the other directions, the cell hardly reacted. Overall, we noted a tendency toward lower DSGC responses in quadruple KO compared to WT mice (Figures 5E and 5F). However, statistical analyses revealed comparable DSGC responses in both genotypes (p > 0.05).

### Imbalance of inhibitory and excitatory synaptic signaling in the quadruple KO retina

**Figure 6:**
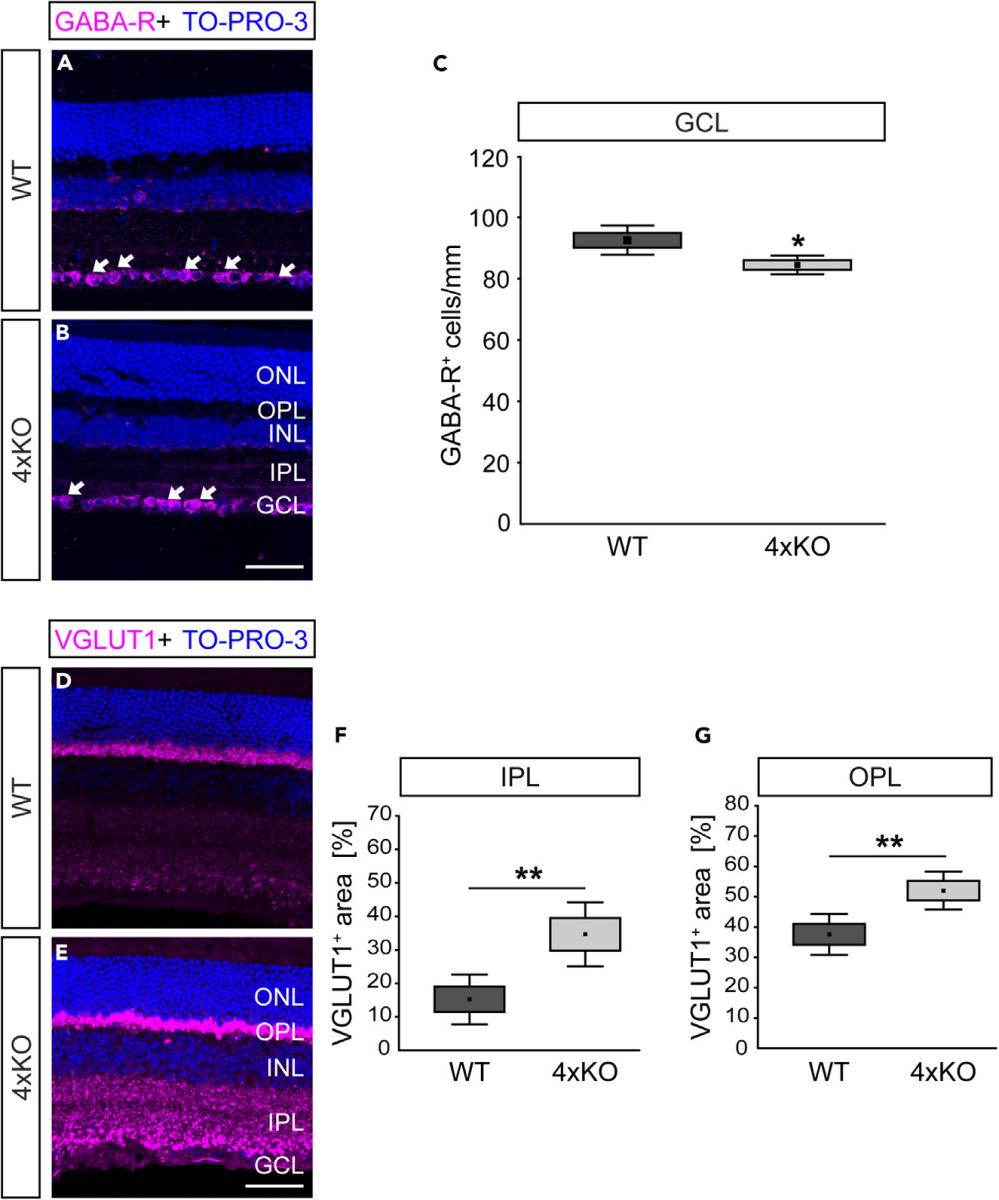
Imbalance of inhibitory and excitatory synaptic signaling in the quadruple KO retina (A and B) Representative images of retinal sections of WT and quadruple KO mice, which were stained using anti-GABA-R (GABA_A_ receptor α2, magenta). Cell nuclei were stained with TO-PRO-3 (blue). (C) Quantification revealed a significantly reduced number of GABA-R^+^ cells in the GCL of quadruple KO mice (p < 0.05). (D and E) Exemplary images of anti-VGLUT1 (magenta) stained retinal sections of WT and quadruple KO mice. (F and G) Quadruple KO mice showed a significantly enhanced excitatory VGLUT1 staining area in the plexiform layers (IPL and OPL). Cell nuclei were stained with TO-PRO-3 (blue). Scale bars: 50 μm. ∗p < 0.05, ∗∗p < 0.01; N = 8. GABA-R, γ-aminobutyric acid A receptor α2, GCL, ganglion cell layer; INL, inner nuclear layer; 4xKO, quadruple knockout; VGLUT1, vesicular glutamate transporter 1; WT, wild-type. Data are shown as mean ± SEM ± SD.

SACs generate direction-selective output of GABA to provide critical inhibition of receptive field properties of DSGCs. As previously shown, GABA_A_ receptors containing the α2 subunit are critical for direction-selective inhibition, and various RGC subtypes, including DSGCs, were previously described to express GABA_A_ receptor α2^+^.^26^^,^^45^ Interestingly, our analyses revealed that quadruple KO mice exhibited a significantly reduced number of inhibitory GABA_A_ receptor α2^+^ cells in the GCL, which might indicate a loss of DSGCs (WT: 92.5 ± 2.4 cells/mm; quadruple KO: 84.5 ± 1.6 cells/mm, p < 0.02, Figures 6A–6C). Since no change in the GABA_A_ receptor α2 signal was noted per counterstained cell (Figures S5A–S5E). Additionally, we performed signal analysis on GABA, which revealed a significantly reduced GABA-positive area in the IPL of quadruple KO compared with WT mice (Figures S6A–S6D).

Inhibition is essential to counterbalance excitatory neurotransmission. Excitatory glutamatergic neurotransmission depends on vesicular glutamate transporters (VGLUTs), which segregate glutamate into synaptic vesicles. As previously shown, VGLUT1 is required for photoreceptor signaling to second- and third-order neurons but not for intrinsic visual functions.^46^ The immunoreactivity pattern of VGLUT1 is restricted to photoreceptor and BC terminals, the principle glutamatergic neurons in the retina.^47^^,^^48^

To comparatively assess VGLUT1 staining in WT and quadruple KO retinae, retinal cross-sections were stained with anti-VGLUT1 antibodies. Representative images show a specific synapse-associated VGLUT1 staining in both plexiform layers (Figures 6D and 6E). However, as validated by quantification, quadruple KO mice showed a significantly larger VGLUT1 staining area in the IPL compared with WT mice (WT: 13.9 ± 8.6% VGLUT1^+^ area, quadruple KO: 37.2 ± 10.5% VGLUT1^+^ area, p < 0.001, Figure 6F). In addition, we found a significantly increased VGLUT1^+^ area in the OPL of quadruple KO in comparison to WT mice (WT: 37.6 ± 9.8% VGlut1^+^ area vs. quadruple KO: 52.0 ± 9.0% VGLUT1^+^ area, p = 0.008; Figure 6G), pointing to an increased excitatory signaling. Taken together, our findings suggest quadruple KO mice exhibit an altered balance of retinal excitatory/inhibitory neurotransmission *in vivo*.

### Global gene expression changes in the quadruple KO retina

To obtain a more detailed picture on global gene expression changes in the adult quadruple KO retina, we performed NGS analyses. Our genomic exon analyses unveiled a distinct allele loss of *Bcan*, *Ncan*, *Tnc*, and *Tnr* in quadruple KO mice (Figures S7A–S7D). Furthermore, western blot analyses showed the presence of all four ECM proteins in the P12 WT retina, whereas these proteins were not detected in the quadruple KO tissue (Figures S8A–S8D).

**Figure 7:**
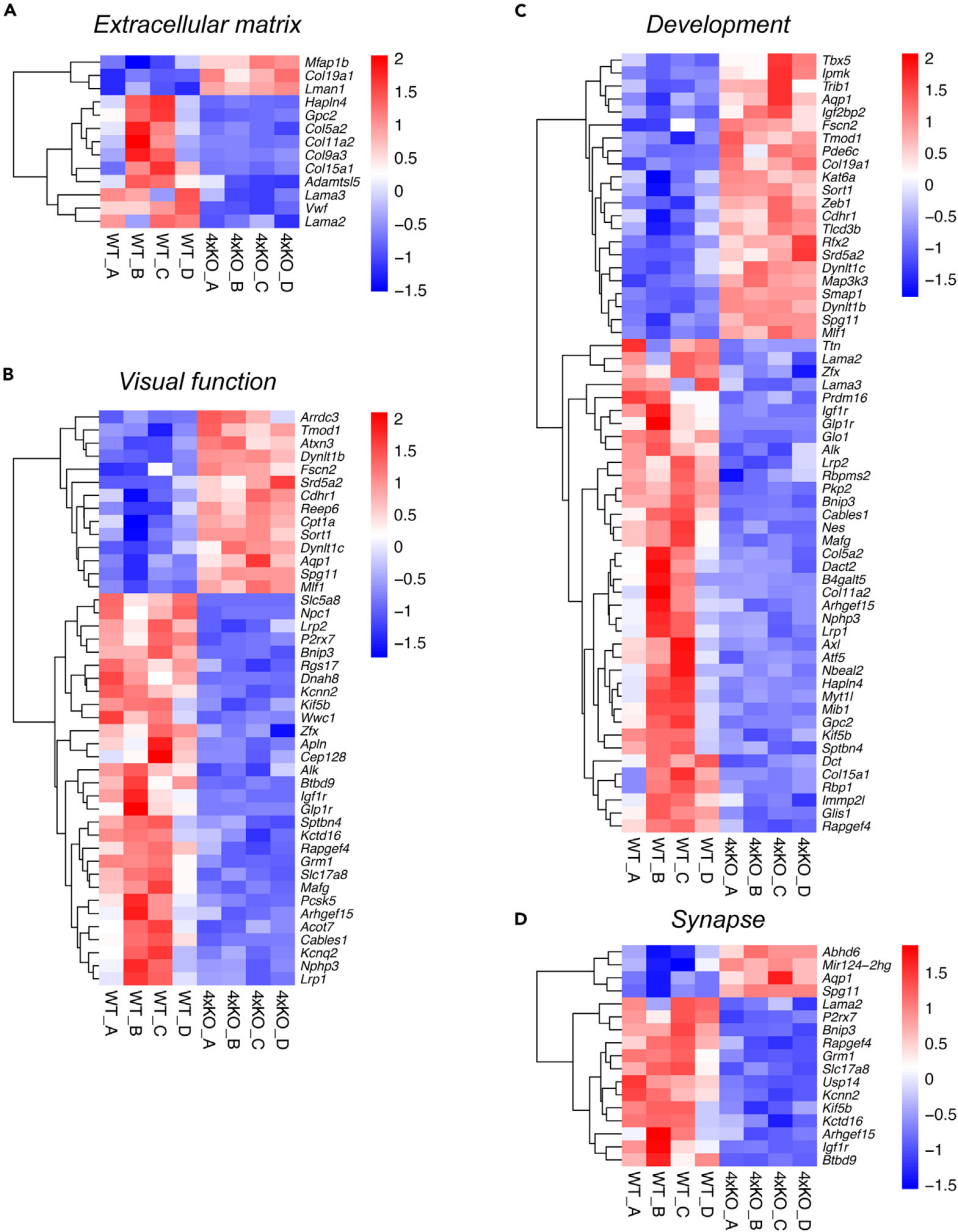
NGS revealed global gene expression changes in the quadruple KO retina (A–D) NGS revealed a different gene expression pattern in the quadruple KO compared with the WT mouse. The heatmaps include significantly regulated genes that belong to the three enriched GOs in function groups of interest (A) *ECM*, (B) *Visual function*, and (C) *Development* as well as for the selected GOs in function group of interest (D) *Synapse*. Heatmaps present the normalized expression (log2 scale) of significantly regulated genes (adjusted p value < 0.05) annotated with GO terms for the four groups of interest. The expression change of these genes is indicated by the color shift from blue to red. Thus, downregulated genes are illustrated in blue, whereas upregulated genes are depicted in red. N = 4xKO, quadruple knockout; WT, wild-type.

Subsequent bioinformatic analyses identified a total of 263 differentially expressed genes (adjusted p < 0.05) of which 142 genes were downregulated and 121 genes were upregulated in the quadruple KO compared with the WT retina (Table S2). The heatmap and volcano plot illustrate the identified significant differentially expressed genes (Figures S9A and S9B). Additionally, heatmaps show row *Z* score scaled expression values of regulated genes (adjusted p < 0.05), which belong to enriched Gene Ontologies (GOs) in three function groups of interest: *Extracellular matrix* (13 genes), *Visual function* (44 genes), and *Development* (60 genes) as well as the selected GOs in function group S*ynapse* (17 genes) (Figures 7A–7D).

Interestingly, we detected a dysregulation of various ECM and ECM-associated genes in the quadruple KO retina. A significant downregulation was noted for *Hapln4* (*hyaluronan and proteoglycan link protein 4*), the ECM-receptor *Gpc2* (*glypican 2*), the *α collagens Col5a*, *Col9a3*, *Col11a2*, and *Col15a1*; *Adamtsl5* (*a disintegrin-like and metalloprotease domain containing thrombospondin type 1 motif-like 5*), as well as the *α laminins Lama2* and *Lama3*. In contrast, *Mfap1b* (*microfibrillar-associated protein 1B*), *Col19a1*, and *Lman 1* (*lectin, mannose-binding 1*) were significantly upregulated in the quadruple KO retina. Besides, we noted a dysregulation (raw p value lower than 0.05) of ECM/ECM-related genes such as *α collagens Col6a1*, *Col1a1*, and *Col2a1*; *laminins Lama1* and *Lamb2*; the ECM-modifying gene *Timp2* (*tissue inhibitor of metalloproteinase 2*); and the ECM-receptors *Itgb4* (*integrin β4*), *Gpc1* (*glypican 1*) as well as *CD44* (cluster of differentiation 44; data not shown).

Additionally, GO term analyses revealed an enrichment of the function group *Visual function*, which includes the upregulation of *Arrdc3* (*arrestin domain containing protein 3*) as well as the AC and Müller-glia-cell-expressed water channel *Aqp1* (*aquaporin 1*).^49^ In contrast, a downregulation was observed for genes such as *Kcnq2* (*potassium voltage-gated channel, subfamily Q, member 2*), *Lrp1* and *Lrp2* (*low-density lipoprotein receptor-related protein 1 and 2*), as well as *Glp1r* (*glucagon-like peptide 1 receptor*)*.*

Among others, enrichment analyses of the function group *Development* showed a downregulation of the RGC-specific gene *Rbpms2* (*RNA binding protein with multiple splicing 2*).^50^

Based on previous knowledge and data of the present work, we were also interested in the dysregulation of genes annotated to the selected function group *Synapse.* Most interestingly, we observed a dysregulation of several synaptic proteins, which included a prominent upregulation of the gene *Abhd6* (*abhydrolase domain containing 6*), the long non-coding RNA *Mir124-2hg*, *Aqp1* (see also GO groups *Visual function* and *Development*), as well as *Spg11* (*spatacsin vesicle trafficking associated*).

A significant downregulation was found, e.g., for the synaptic/synapse-associated genes *Lama2*, *P2rx7* (*purinergic receptor P2X, ligand-gated ion channel 7*), *Bnip3* (*BCL2/adenovirus E1B interacting protein 3*), *Rapgef4* (*Rap guanine nucleotide exchange factor [GEF] 4*), *Grm1* (*glutamate receptor, metabotropic 1*), *Slc17a8 (sodium-dependent inorganic phosphate cotransporter, member 8*; also known as *the vesicular glutamate transporter 3/VGLUT3*), *Kcnn2* (*potassium intermediate/small conductance calcium-activated channel, subfamily B, member2*), *Kctd16* (*potassium channel tetramerization domain containing 16*), *Igf1r* (insulin-like growth factor I receptor), and *Btbd9* (*BTB [POZ] domain containing 9*).

Collectively, we noted an extensive ECM remodeling and synaptic imbalance in quadruple KO mice, which might directly contribute to the observed functional and optomotor deficits.

## Discussion

In the 1960s, direction-selective nerve cells in the retina of vertebrates, which only become activated when a light stimulus completes a specific movement, were first described.^51^ These cells, known today as SACs, play a critical role in inhibitory GABAergic and excitatory cholinergic modulation of DSGC responses, contributing to directional selectivity and the recognition of directional motion.^52^ In our study, we report on the impairment of visual motion processing and loss of cholinergic direction-selective SACs in quadruple KO mice lacking the four ECM proteins Bcan, Ncan, Tnc, and Tnr. A reduced number of SACs in the quadruple KO at P10 suggests that the loss of the ECM constituents already impacts early development of SACs. In this regard, the specification, maturation, migration, and/or survival of SACs might be affected. Accordingly, tenascins and CSPGs have been shown to provide a crucial modulatory environment for those cellular functions.^53^^,^^54^^,^^55^^,^^56^ We cannot exclude that also other cell types are affected by the loss of the four ECM molecules. However, particularly the loss of Tnc expression in SACs might be directly related to a cell intrinsic mechanism. Our analyses in the WT retina revealed robust expression of all four molecules in postnatal stages. In the postnatal retina, release of ACh by SACs is required for proper propagation of spontaneous activity in the form of retinal waves.^43^^,^^57^ Direction selectivity in the retina, however, seems to emerge independently of visual experience and cholinergic waves and thus most likely arises due to complex molecular interactions.^58^ Hence, the four ECM molecules are promising candidates that might contribute to the fine-tuning and establishment of neuronal circuits.

We observed that quadruple KO mice exhibit reduced a- and b-wave amplitudes, suggesting retinal dysfunction. We previously noted normal ERG responses in *Tnc* KO mice.^59^^,^^60^ Thus, we propose that the combined loss of four ECM molecules or alternatively the combined or single loss of Bcan, Ncan, and Tnr may contribute to impaired ERG responses and retina function. Interestingly, we found that b-wave responses derived from excitatory glutamatergic rod BCs were most severely affected in the quadruple mutant. Cholinergic feedback to BCs enhances direction-selective signaling in postsynaptic SACs and DSGCs.^61^ Therefore, the loss of cholinergic SACs and the reduced cholinergic feedback to rod BCs could explain the severe impairment of b-wave responses. The effects of the loss of ECM molecules on retinal functionality of sensory tissues such as the retina have been poorly described. So far, the ECM in tissues is rather known for its role in homeostasis and the organization of synapses.

We noted an imbalance of inhibitory GABAergic and excitatory glutamatergic signaling in the quadruple KO retina *in vivo*. Therefore, forthcoming experiments should prioritize the investigation of synaptic alterations by marker analyses and employing techniques such as super-resolution microscopy and electrophysiology. Previous studies documented that the elimination of the four ECM proteins alters the ratio of excitatory and inhibitory hippocampal synapses *in vitro*.^30^ Depletion experiments indicate that the ECM preserves the balanced state of neuronal networks by stabilizing inhibitory synapses.^1^ Of note, an imbalance of GABA/glutamate has been suggested in albino rats with missing optokinetic nystagmus.^62^^,^^63^ Our findings suggest that mutual interactions of the matrisome regulate the delicate balance between GABAergic inhibition, glutamatergic excitation, and visual motion processing *in vivo*. Tnc and Tnr bind Ncan and Bcan, respectively, with a high affinity.^64^^,^^65^ In astrocytes, Tnr modulates the uptake of glutamate.^66^ A reduced GABAergic inhibition was found in the hippocampus after ablation of Tnr.^18^ Based on these reports, we hypothesize that the cooperation of four matrix molecules is crucial for a balanced synaptic signaling. In that context, Tnr appears to exert a regulatory influence on GABAergic inhibition. Recently, it was reported that the neural ECM preserves the equilibrium of neuronal networks by stabilizing inhibitory synapses.^32^ In this perspective, the reduction of the ECM might directly contribute to a weakening of inhibitory synapses.

Our analyses revealed a reduced number of GABA-R^+^ cells in the KO retina, which might indicate a loss of DSGCs. Therefore, future analysis should focus on the characterization of the specific affected DSGCs and/or DSGC subpopulation. However, the MEA measurements revealed no significant different DSGC responses in quadruple KO mice compared with WT ones, which might be explained by the limited sensitivity of MEA measurements and/or compensatory mechanisms. In this regard, it is worth considering that various other ECM constituents, including laminins, collagens, several ECM receptors, and ECM-modifying molecules were found dysregulated in our NGS analyses. Furthermore, the loss of SACs might be compensated by remaining SACs, which provide sufficient input to DSGCs.

In the CNS, tenascins exert a strong influence on synaptic structure and function.^4^^,^^5^ Thus, the absence of Tnc and Tnr in the quadruple KO could be one cause of impaired visual processing. To trace these limitations back to individual Tnc or Tnr genes, OMR measurements were carried out in single KO mice. When measured at both low and high velocities, *Tnc* KO mice showed a significantly reduced number of head movements compared with the WT. In contrast, in the *Tnr* KO, the number of head movements was comparable to the WT at low velocities in either direction. At higher velocities, however, the number of head movements was also reduced in the *Tnr* KO. At slow velocities counterclockwise, a significant reduction of head movements was observed in *Tnc* KO in comparison to *Tnr* KO mice. These results indicate that the elimination of Tnc leads to limitations in visual processing at low and high velocities, whereas the *Tnr* deficiency mitigates processing only at high velocities, possibly due to compensatory effects. Yet, both the ablation of Tnc and Tnr impaired visual motion processing. The phenotype was accentuated in quadruple KO mice, where the number of head movements was significantly diminished in contrast to both *Tnc* and *Tnr* KO mice. This strongly suggests that the loss of four ECM molecules intensified the limitations of visual motion processing that appeared to a milder degree in the single KOs. Visually triggered compensatory head movements (optomotor responses) or eye movements (optokinetic responses) are widely employed in experimental mouse models to study abnormalities and pathological conditions and assess the effectiveness of therapeutic interventions. Kretschmer et al. found that both optomotor and optokinetic responses provide very similar outcomes when assessing visual acuity in mice.^38^ Moreover, the application of a head tracking algorithm allows for a significantly more precise analysis of head angle determination and reveals individual head retractions, analogous to the saccadic eye movements observed during optokinetic nystagmus. When interpreting the results, it should be noted that OMR measurements and analyses of head movement in response to visual input constitute a first-line visual behavior test, which does not convey the same level of information as OKR measurements regarding visual functions. Nevertheless, in the context of the impaired ERG measurements in quadruple KO mutants, our findings unequivocally indicate functional visual impairments in visuomotor processes. However, it is important to note that those tests may not fully reflect visual behavior, and future visual acuity tests may provide more comprehensive insights. The observed anomalies of the ECM mutants may reflect gene-dosage effects of the matrisome and reveal cooperative synergism of the four ECM molecules. This may be based on molecular interactions as well as remote impact on gene regulation in the KO lines. The establishment of the precise pathways involved will require further investigations.

The ablation of SACs has been shown to decrease direction selectivity in retinal ON-OFF DSGCs *in vivo*.^67^ ON-OFF DSGCs primarily project to the lateral geniculate nucleus (LGN) and the primary visual cortex (area V1 for visual motion integration).^68^^,^^69^^,^^70^^,^^71^ Thus, it is reasonable to assume that deficiency of optomotor behavior observed in quadruple KO mice could potentially be explained by an impaired (sub-) cortical processing. In our recent study, we reported on PNN-associated ECM changes in the quadruple KO V1, which could directly affect optomotor behavior.^31^ Notably, a distinct PNN-associated ECM expression was noted in the LGN, which may also play a role in visual motion processing.^72^ Therefore, it is possible that cumulative changes in the ECM across various brain regions critical for visual motion computation could additionally contribute to the optomotor behavior deficiency observed in the quadruple KO model.

The causes for the restrictions of visual movement processing could also be disturbances in the synaptic circuitry and the excitation/inhibition ratio of the retina. Interestingly, the NGS analyses are in accordance with the hypothesis of a synaptic imbalance in the quadruple KO retina, as a dysregulation of several synaptic candidates was registered. For example, we noted a downregulation of the RNA-binding protein *Rbpms2/Hermes*, a crucial modulator of synapse density as well as axon arbor formation in RGCs, which also influences optomotor processing.^50^ We observed a downregulation of the *Kcnq2* gene, which is involved in the proper function of potassium channels in the brain. The functional importance of *Kcnq2* becomes particularly obvious under pathological conditions. Thus, alterations of KCNQ2 have been associated with seizures, autism as well as cognitive and developmental disabilities.^73^ In a recent study, *Kcnq2* was presented as an important downstream regulator of ketamine in hippocampal glutamatergic neurons.^74^ Also, we noted a downregulation of the G-protein-coupled receptor *Glp1r* (*glucagon-like peptide-1 receptor*). The *Glp1r* agonist exendin-4 suppresses GABAR-mediated light-evoked inhibitory postsynaptic currents in RGCs.^75^ We also found a downregulation of the *vesicular glutamate transporter 3/VGLUT3* (*Slc17a8*)*.* Interestingly, VGLUT3-expressing ACs provide excitatory input to ON-OFF and ON DSGCs and a subpopulation of W3 RGCs but not SACs.^76^ Therefore, the ECM loss in quadruple mutants might also impact SAC-independent excitatory glutamatergic signaling. In this regard, we observed downregulation of the *metabotropic glutamate receptor 1/Grm1.*^77^^,^^78^ Furthermore, we noted a downregulation of *Lrp1*, an important modulator of the ECM.^79^ Interestingly, deletion of *Lrp1* from astrocytes in a hippocampal neuron co-culture model decreased neuronal network activity and influenced the proportion of pre- and postsynaptic structures.^80^ In summary, we noted an extensive ECM remodeling and synaptic imbalance in quadruple mutant mice, which contribute to the impaired visual function and optomotor behavior.

A groundbreaking discovery had revealed that CSPG degradation by injection of chondroitinase ABC into the visual cortex of adult rats leads to a reactivation of ocular dominance plasticity.^81^ This observation provided evidence that the mature ECM inhibits experience-dependent plasticity and plays a central role in regulating visual input processing in the visual cortex. Conditions of degeneration such as in glaucoma are associated with complex ECM remodeling in the retina.^33^^,^^59^^,^^60^ However, these studies mainly focused on structural ECM changes concurrent with visual impairment caused by disease, rather than on the direct effect of ECM on visual function. Hence, our study provides crucial evidence for an impairment of sensorimotor integration in a neuronal subsystem as direct consequence of ECM deficiency.

### Limitations of the study

A limitation of our study is that the quadruple KO model provides insight into the cooperative effects of several ECM proteins but does not circumscribe the individual roles of the singular ECM molecule regarding the observed deficits in retina function. However, we revealed, yet less pronounced, optomotor deficits in single mutants for *Tnc* and *Tnr*. The relative contributions of the CSPGs Bcan and Ncan as well as the establishment of the pathways involved will require further investigations. Additionally, further visual acuity tests may provide more comprehensive insights into the visual behavior of ECM mutants.

## STAR★Methods

### Key resources table

**Table:** 

REAGENT or RESOURCE	SOURCE	IDENTIFIER
**Antibodies**		

Alexa Fluor 488-AffiniPure Donkey Anti-Goat IgG (H+L)	Jackson ImmunoResearch Labs	Cat# 705-545-147; RRID: AB_2336933
Alexa Fluor 488-AffiniPure Goat Anti-Mouse IgG + IgM (H+L)	Jackson ImmunoResearch Labs	Cat# 115-545-044; RRID: AB_2338844
Alexa Fluor 488-AffiniPure Goat Anti-Rabbit IgG (H+L)	Jackson ImmunoResearch Labs	Cat# 111-545-045; RRID: AB_2338049
Anti-Bcan (guinea pig)	Seidenbecher et al.^83^	N/A
Anti-Bcan (mouse)	BD Biosciences	RRID: AB_398211
Anti-ChAT	Millipore	Cat# AB144; RRID: AB_11212843
Anti-DIG Fab fragment antibody, AP-conjugated	Roche Diagnostics GmbH	Cat# 11093274910; RRID: AB_514497
Anti-GABA	Sigma-Aldrich	Cat# A 2052; RRID: AB-477652
Anti-GABA_A_ receptor α2	Synaptic Systems GmbH	Cat# 224 103; RRID: AB_2108839
Anti-Ncan (rabbit)	Rauch et al.^84^	N/A
Anti-Ncan (sheep)	R&D Systems	RRID: AB_2149717
Anti-Tnc	Faissner and Kruse^85^	N/A
Anti-Tnr	Rathjen et al.^86^	N/A
Anti-VGLUT1	Sigma-Aldrich	Cat# V0389; RRID: AB_261840
Biotin-SP-AffiniPure Goat Anti-Guinea Pig IgG (H+L)	Jackson ImmunoResearch Labs	Cat# 106-065-003; RRID: AB_2337410
Peroxidase-AffiniPure Goat Anti-Mouse IgG + IgM (H+L)	Jackson ImmunoResearch Labs	RRID: AB_2338505
Peroxidase-AffiniPure Goat Anti-Rabbit IgG (H+L)	Jackson ImmunoResearch Labs	RRID: AB_2307391
Peroxidase-AffiniPure Donkey Anti-Sheep IgG (H+L)	Jackson ImmunoResearch Labs	RRID: AB_2340710

**Bacterial and virus strains**		

One Shot™ TOP10 chemically competent E. coli cells	Thermo Fisher Scientific	Cat# C404003

**Biological samples**		

WT and quadruple KO retina tissue	This work	N/A

**Chemicals, peptides, and recombinant proteins**		

BamHI	Thermo Fisher Scientific	Cat# ER0051
BCIP	Roche Diagnostics GmbH	Cat# 11383221001
Blocking reagent	Roche Diagnostics GmbH	Cat# 11096176001
Cy2-Streptavidin	Jackson ImmunoResearch Labs	Cat# 016-220-084
EcoRI	Thermo Fisher Scientific	Cat# ER0271
HindIII	Thermo Fisher Scientific	Cat# ER0501
NBT	Roche Diagnostics GmbH	Cat# 11383213001
TO-PRO™-3 iodide	Thermo Fisher Scientific	Cat# T3605
XhoI	Thermo Fisher Scientific	Cat# ER0691

**Critical commercial assays**		

BCA Protein Assay Kit	Thermo Fisher Scientific	Cat# 23225
cDNA synthesis kit	Fermentas GmbH	Cat# K1612
DIG RNA Labeling Mix	Roche Diagnostics GmbH	Cat# 26591020
DirectPCR® Lysis Reagent Tail	VWR	Cat# 31-101-T
DNaseI (Rnase-free)	Roche Diagnostics GmbH	Cat# 04716728001
JumpStart™ Tag DNA polymerase	Sigma-Aldrich	Cat# D9307
NEBNext Ultra II Directional Total RNA Library Prep Kit for Illumina	NEB	Cat# E7760S/L
Precision Plus Protein Standards Dual Color Standards	Bio-Rad Laboratories GmbH	Cat# 1610374
Protease Inhibitor Cocktail	Sigma-Aldrich	Cat# P2714
Proteinase K	Roth	Cat# 7528.3
RNeasy® Mini Kit	Qiagen	Cat# 74104
RNeasy® Midi Kit	Qiagen	Cat# 12143
SP6 RNA polymerase	Roche Diagnostics GmbH	Cat# EP0131
T7 RNA polymerase	Roche Diagnostics GmbH	Cat# EP0111
TOPO® TA Cloning® Kit	Thermo Fisher Scientific	Cat# 45 - 0640
QIAEX II® Gel Extraction Kit	Qiagen	Cat# 20021

**Deposited data**		

RNA-seq data	GEO	GEO: GSE230425

**Experimental models: Organisms/strains**		

129S2/SvPasCrl mice	Charles River Laboratories	RRID: IMSR_CRL:287
Tnc KO mice	Forsberg et al.^87^; Czopka et al.^88^	N/A
Tnr KO mice	Weber et al.^89^; Czopka et al.^88^	N/A
Quadruple KO mice	Rauch, U. et al.^27^	N/A

**Oligonucleotides**		

For primer pairs, see Tables S3 and S4	Sigma-Aldrich	Tables S3 and S4

**Recombinant DNA**		

pCRTM2.1-TOPO vector	Thermo Fisher Scientific	Cat# 45 - 0640

**Software and algorithms**		

Adobe Illustrator	Adobe	http://www.adobe.com/products/illustrator.html; RRID: SCR_010279
Adobe Photoshop	Adobe	https://www.adobe.com/products/photoshop.html; RRID: SCR_014199
AxioVision Imaging System	Carl Zeiss	RRID: SCR_002677
Black ZEN software	Carl Zeiss	RRID: SCR_018163
Coral Paint Shop Pro	Coral Corporation	N/A
DESeq2 v.1.32.0 framework	Love et al.^90^	https://bioconductor.org/packages/release/bioc/html/DESeq2.html; RRID: SCR_015687
ERGView 4.380R	OcuScience	N/A
ImageJ	National Institutes of Health	https://imagej.nih.gov/ij/index.html; RRID: SCR_003070
MATLAB	The MathWorks	http://www.mathworks.com/products/matlab/RRID:SCR_001622
Offline Sorter	Plexon Inc	http://www.plexon.com/products/offline-sorter; RRID: SCR_000012
R library gprofiler2	Kolberg et al.^91^	https://biit.cs.ut.ee/gprofiler/page/r; RRID: SCR_018190
Salmon v1.5.2	Patro et al.^92^	https://combine-lab.github.io/salmon/; RRID: SCR_017036
STATISTICA V13.3	StatSoft	RRID: SCR_014213

**Other**		

60MEA200/30IR-ITO	MultiChannel Systems	N/A
Axioplan2	Carl Zeiss	N/A
BioSpectrometer	Eppendorf	N/A
CM3050 S cryostat	Leica	N/A
HMsERG system	OcuScience	N/A
Illumina NextSeq500	Illumina	N/A
LSM 510 META	Carl Zeiss	N/A
MicroChemi Chemiluminescence Reader	Biostep	N/A
TonoLab	Icare	N/A
Quantus Fluorometer	Promega	N/A

### Resource availability

#### Lead contact

Further information and requests for resources and reagents should be directed to and will be fulfilled by the lead contact Andreas Faissner. Email: andreas.faissner@rub.de.

#### Materials availability

This study did not generate new unique reagents.

#### Data and code availability

• The RNA sequencing data have been deposited at the NCBI Gene Expression Omnibus (GEO) database (https://www.ncbi.nlm.nih.gov/geo/) and are publicly available as of the date of publication through the GEO accession number GSE230425.
• This study did not generate any original code.
• Any additional information required to reanalyze the data reported in this paper is available from the lead contact upon request.

### Experimental model and study participant details

#### Animals

Mouse breeding colonies were maintained at the animal facility of the Faculty of Biology and Biotechnology, Ruhr University Bochum. Animals were housed under environmentally controlled lighting conditions (12-hour light-dark cycle) with access to chow and water *ad libitum*. Adult quadruple KO mice, generated by Rauch and colleagues through cross-breeding of the described single KO mutant mouse lines, combining the transgenic KO constructs of *Bcan, Ncan, Tnc* and *Tnr*^16^^,^^27^^,^^87^^,^^89^^,^^93^ as well as mice only carrying the *Tnc* KO construct or the *Tnr* KO construct were compared to WT mice from the inbred background strain 129S2/SvPasCrl (Charles River Laboratories, Sulzfeld, Germany). Animals were mated overnight, and females were checked in the morning for the presence of a vaginal plug, which corresponded to embryonic day 0.5 (E0.5). For analyses, animals from the postnatal (P) stages P0, P4, P8, and P12 as well as adult mice of both sexes were euthanized by cervical decapitation or dislocation.

#### Animal study approval

The animal studies conducted in this work followed the guidelines of the Association for Research in Vision and Ophthalmology (ARVO) statement for the use of animals in ophthalmic and vision research. The experiments were performed in compliance with the German law (§15 TierSchG) and approved by the animal protection committee (Landesamt für Natur, Umwelt und Verbraucherschutz, Recklinghausen, North Rhine-Westphalia Germany; file number 84-02.04.2013.A291). The study was supervised by the animal welfare commissioner of the Ruhr University Bochum and the clinics facility for animal welfare (Einrichtung für Tierschutz, Tierärztlichen Dienst und Labortierkunde) of the University Tübingen. All efforts were made to minimize the number of animals used and their suffering.

### Method details

#### Genotyping

For the isolation of genomic DNA, mouse tail biopsies were lysed overnight at 55°C in DirectPCR® Lysis Reagent Tail (VWR, Radnor, PA USA) containing 0.2 mg/ml proteinase K (Roth, Karlsruhe, Germany). The following day, samples were incubated for 45 minutes at 55°C. For genotyping, PCR analyses were performed using JumpStart™ Taq DNA polymerase and primer pairs (Sigma-Aldrich, St. Louis, MO, USA) listed in Table S3. The following PCR conditions were used: 94°C for 2 minutes 40 seconds (initial denaturation), 37 cycles of 94°C for 30 seconds (denaturation), 56-60°C for 30 seconds (annealing), and 72°C for 50 seconds followed by 72°C for 5 minutes (final elongation). Following agarose gel electrophoresis (gel electrophoresis unit pharmacia biotech, Amersham, Freiburg, Germany) samples were documented under UV light in a gel documentation system (LFT Labortechnik GmbH & Co.KG, Wasserburg, Germany).

#### Intraocular pressure measurements

Intraocular pressure (IOP) measurements in quadruple KO and WT mice of both genders (16 weeks of age) were performed with a rodent rebound tonometer (TonoLab, Icare, Vantaa, Finland) as previously described.^94^

#### Electroretinogram recordings

Scotopic full-field flash electroretinogram (ERG) recordings were done with a HMsERG system (OcuScience, Henderson, NV, USA) as described previously.^94^ Quadruple KO and WT mice of both genders (16 weeks of age) were dark-adapted overnight. For measurements, mice were hold under dim red light and anesthetized by an intraperitoneal injection of a ketamine-xylazine cocktail (120/16 mg/kg body weight). Afterward, eyes were topically anesthetized with Oxybuprocaine hydrochloride (Novesine Stulln® 4 mg/ml, Pharma Stulln, Stulln, Germany) and the pupil was dilated with Tropicamide (Mydriaticum Stulln® 5 mg/ml, Pharma Stulln, Stulln, Germany). Scotopic flash series were recorded at 0.1, 0.3, 1.0, 3.0, 10, and 25 cd/m^2^. Then, electrical potentials were analyzed by the ERGView 4.380R software (OcuScience). A 150 Hz low-pass filter was applied and a/b-wave amplitudes and implicit times were evaluated. The a-wave amplitudes were measured from the pre-stimulus baseline up to the a-wave, while the b-waves were measured from the a-wave to the b-wave peak. The a-wave implicit time was calculated from the onset of the light flash stimulus to the peak of the a-wave, while the implicit time of the b-wave was measured from the peak of the a-wave to the peak of the b-wave.

#### Measurement of optomotor responses

To induce the optomotor response (OMR), mice were placed in a rotating cylinder (30 cm ø) with a vertical black and white stripe pattern. The stripes were 2 cm wide. Optimal velocities to elicit the OMR were 20^°^/second and 50^°^/second and rotation was done clockwise (CW) and counterclockwise (CCW). OMR was assessed by counting the fast-returning movement of the head after following the stripe pattern (defined as number of head movements). Analyses were done in male and female WT and quadruple KO as well as *Tnc* and *Tnr* single KO mice at 16 weeks of age.

#### Multielectrode array

Microelectrode array (MEA) recordings have been reported previously.^95^ To record spiking neural activity of RGCs, dark-adapted quadruple KO and WT retinae were prepared and mounted RGC side down contacting the 60-channel MEA (60MEA200/30IR-ITO; MultiChannel Systems, Reutlingen, Germany). MEA-mounted retinae were superfused with artificial cerebrospinal fluid (ACSF) solution (125 mM NaCl, 2.5 mM KCl, 2 mM CaCl_2_, 1 mM MgCl_2_, 1.25 NaH_2_PO_4_, 26 mM NaHCO_3_ and 20 mM glucose) at a rate of 2.5 ml/minute at 33°C and left to recover for at least 30 minutes before recording. Extracellular voltage signals were recorded with equipment from MultiChannel Systems, processed offline to isolate spike trains using Offline Sorter (Plexon Inc., Dallas, TX, USA), and analyzed with custom scripts in MATLAB (The MathWorks, Natick, MA, USA). To probe for direction selectivity, we presented moving bar light stimuli, adapted from the visual stimulation set described by Baden and colleagues.^96^ In brief, stimuli were designed and delivered through QDSpy. The moving bar stimulus was represented as a white bar (300 x 1000 μm) drifting along its long axis in a sequence of positions covering the MEA and repeated at 8 different directions in sequence to quantify direction-selectivity.

#### Tissue collection for RT-PCR, immunohistochemistry and *in-situ* hybridization

For RNA extraction, retinae were isolated, pooled, snap frozen in liquid nitrogen and stored at -80°C until RNA extraction. For immunohistochemistry and *in-situ* hybridization of retinal sections, whole embryo heads, postnatal heads, and adult eyes were fixed in 4% paraformaldehyde (PFA, Sigma-Aldrich) for 12-24 hours at 4°C and cryo-protected in 30% sucrose (J. T. Baker, Deventer, NL). Tissue was embedded in Jung TISSUE FREEZING MEDIUM™ (Leica Instruments GmbH, St. Leon-Rot, Germany), sectioned in horizontal planes of 16 μm using a cryostat (CM3050 S, Leica, Bensheim, Germany), and collected onto Superfrost plus object slides (Menzel-Gläser, Braunschweig, Germany). Thereafter, slides were stored at -80°C until further processing. For the preparation of retinal flat-mounts, eyes were enucleated and fixed in 4% PFA at 4°C for 1 hour. Then, the retinae were dissected, post-fixed in 4% PFA for 5 minutes, and washed in 1 x phosphate buffered saline (1 x PBS) until immunohistochemical staining.

#### RNA preparation, cDNA synthesis and reverse-transcription polymerase chain reaction

Total RNA was extracted using the RNeasy® Mini or Midi kit (Qiagen, Hilden, Germany) and digested with Rnase-free DNaseI (Roche, Mannheim, Germany) according to the manufacturer’s instructions. The quality and quantity of RNA was assessed photometrically using the BioSpectrometer® (Eppendorf, Hamburg, Germany). To obtain cDNA, 1 μg of RNA was used for reverse transcription with a cDNA synthesis kit and random hexamer primers (Fermentas GmbH, St. Leon-Rot, Germany). Reverse-transcription polymerase chain reaction (RT-PCR) was performed using 1 μl cDNA using Taq DNA polymerase (JumpStart™ Taq DNA polymerase (Sigma-Aldrich) in a Mastercycler Gradient (Eppendorf, Hamburg, Germany) with primer pairs listed in Table S4 (Sigma-Aldrich). The RT-PCR conditions were as follows: 94°C for 2 minutes 30 seconds (initial denaturation), 25-35 cycles of 94°C for 30 seconds (denaturation), 60°C for 45 seconds (annealing), and 72°C for 60 seconds followed by 72°C for 5 minutes (final elongation). Samples were documented as described above.

#### Next generation sequencing and bioinformatic analyses

For next generation sequencing (NGS), total RNA was isolated from adult WT and quadruple KO retinae (N = 4) as described above. The quantity of RNA was analyzed with the Quantus Fluorometer (Promega, Madison, WI, USA). RNA quality control was done with the RNA ScreenTape assay using the 4200 TapeStation system (Agilent Technologies Inc., Santa Clara, CA, USA). An RNA Integrity Number (RIN) of at least 7.8 verified the high quality of all included RNA samples. Sequencing libraries were generated from 450 ng of total RNA using the NEBNext Ultra II Directional Total RNA Library Prep Kit for Illumina as described by the manufacturer (NEB, Ipswich, MA, USA). The libraries were run on an Illumina NextSeq500 platform using the High Output 150 cycles Kit (Illumina, San Diego, CA, USA). FASTQ files were generated using bcl2fastq (Illumina). To facilitate reproducible analyses, samples were processed using the publicly available nf-core/RNA-seq pipeline version 3.5^97^ implemented in Nextflow 21.10.6^98^ using Docker 20.10.12^99^ with the minimal command. In brief, lane-level reads were trimmed with Trim Galore 0.6.7^100^ and aligned to the mouse genome (GRCm39) using STAR 2.7.9a.^101^ Gene-level and transcript-level quantification was done by Salmon v1.5.2.^92^ Differential expression analyses were performed using custom scripts in R version 4.1.1 with the DESeq2 v.1.32.0 framework.^90^ Genes were considered differentially expressed if the adjusted p-value was < 0.05. The 142 downregulated genes and 121 upregulated genes were further implemented in a gene ontology analyses with R library gprofiler2^91^ using gost function, correction_method = “fdr” and significant = TRUE. Gene ontology terms were considered significant with a false discovery rate < 0.05. As sources, we used Gene Ontology (GO or by branch GO:MF, GO:BP, GO:CC), Kyoto Encyclopedia of Genes and Genomes (KEGG), Reactome (REAC), WikiPathways (WP), and miRTarBase (MIRNA). The volcano plot and heatmaps were constructed with the R package ggplot2. The Integrative Genomics Viewer was used for genomic analyses of quadruple KO alleles (Figure S7).^102^

#### In-situ hybridization

The *in-situ* hybridization (ISH) procedure was performed following a protocol described by N. P. Pringle and W. D. Richardson (Wolfson Institute for Biomedical Research, London, UK). For the design of *in-situ* riboprobes, cDNAs of *Bcan*, *Ncan*, *Tnc,* and *Tnr* were amplified with the ISH primers described above (Table S4). Using the TOPO™ TA Cloning™ Kit with One Shot™ TOP10 chemically competent E. coli cells (Thermo Fisher Scientific, Invitrogen, Waltham, MA, USA), gel purified (QIAEX II® Gel Extraction Kit, Qiagen, Hilden Germany) RT-PCR products were cloned into the pCRTM2.1-TOPO vector (Thermo Fisher Scientific, Invitrogen) containing promotors for SP6 and T7 RNA polymerases (Roche Diagnostics GmbH, Mannheim, Germany). The generation of *Tnc* and *Tnr* riboprobes was described previously.^88^ After linearization with the respective restriction enzymes BamHI, EcoRI, HindIII, or XhoI (Thermo Fisher Scientific, Waltham, MA, USA), digoxigenin (DIG)-labeled antisense and control sense RNA probes were made by *in vitro* transcription using the DIG RNA Labeling Mix (Roche Diagnostics GmbH) following the manufacturer’s protocol. For hybridization, DIG-labeled RNA probes were diluted 1:1,000 in Rnase-free hybridization buffer (1 x salts, 50% formamide, 0.1 mg/ml yeast tRNA, 10% dextran sulfate, and 1 x Denhardt`s) heated to 75°C for 10 minutes, applied to retinal slices and incubated in a humified chamber (2 x saline sodium citrate (SSC) and 50% formamide) at 65°C overnight. The next day, slices were washed twice in washing buffer containing 1 x SSC, 50% formamide and 0.1% Tween-20 at 65°C for 30 minutes. Then, sections were washed twice in MABT buffer (100 mM maleic acid, 150 mM NaCl and 0.1% Tween-20, pH 7.5) at room temperature for 5 minutes. After stringent washing, retinal sections were transferred in a humified (H_2_O) chamber and incubated in blocking solution containing 2% blocking reagent (Roche Diagnostics GmbH, Mannheim, Germany) and 10% heat inactivated sheep serum in MABT buffer for 1 hour at room temperature. Afterward, slices were treated with alkaline phosphatase (AP)-conjugated anti-digoxigenin (DIG) Fab fragment antibody (sheep, 1:1,500, Roche Diagnostics GmbH) in blocking solution at 4°C overnight. Following antibody incubation, slides were washed three times in MABT buffer for 10 minutes and incubated twice in pre-developing buffer containing 100 mM Tris, pH 9.8, 100 mM NaCl, and 50 mM MgCl_2_ for 15 minutes at room temperature. Hybridization signals were visualized by applying NBT/BCIP (4-Nitro blue tetrazolium chloride/1.5-bromo-4-chloro-3-indolyl-phophate, 1 mg/ml, Roche Diagnostics GmbH) developing solution (pre-developing solution and 5% polyvinyl alcohol, pH 9.5) for 2-4 hours at 37°C. To finish the staining reaction, retinal slides were washed in H_2_O, mounted with ImmuMount (Thermo Scientific Shandon™, Waltham, MA, USA) and stored at 4°C.

#### Immunohistochemistry

Slides with retinal cross-sections were washed in 1 x PBS and incubated in blocking solution containing 1-3% normal goat or donkey serum (Dianova GmbH, Hamburg, Germany), 1% bovine serum albumin (BSA, AppliChem GmbH, Darmstadt, Germany) and 0.5% Triton™-X-100 (Sigma-Aldrich, St. Louis, MO, USA) in 1 x PBS at room temperature for 1 hour. Then, primary antibodies were diluted in blocking solution and incubated at room temperature overnight. The following primary antibodies were used in this study: anti-Bcan (guinea pig, 1:250,^83^), anti-ChAT (goat, 1:200, Millipore, Billerica, MA, USA), anti-GABA (rabbit, 1:200, Sigma-Aldrich), anti-GABA_A_ receptor α2 (rabbit, 1:400, Synaptic Systems GmbH, Göttingen, Germany), anti-Tnc (batch KAF14/1, rabbit, 1:300,^85^ anti-Ncan (rabbit, 1:300;^84^), anti-Tnr (anti-restrictin, clone 23-14, mouse, 1:100,^86^), and anti-VGLUT1 (rabbit, 1:400, Sigma-Aldrich). Following further washing in 1 x PBS, sections were incubated in blocking solution without Triton™-X-100 containing appropriate secondary antibodies (donkey anti-goat, 1:300, Alexa Fluor 488, Jackson ImmunoResearch, West Grove, PA, USA; goat anti-mouse, 1:250, Alexa Fluor 488, Jackson ImmunoResearch; goat anti-guinea pig, 1:500, Biotin-SP, Jackson ImmunoResearch; goat anti-rabbit, 1:300, Alexa Fluor 488, Jackson ImmunoResearch). Biotin-Streptavidin immunostaining was detected with Streptavidin Cy2 (1:1,000, Jackson ImmunoResearch). TO-PRO™-3 iodide (1:400, Thermo Fisher Scientific, Invitrogen) was added to visualize cell nuclei. Negative control samples included retinal sections that were subjected to secondary antibody incubation along with TO-PRO™-3 iodide, without prior treatment with primary antibodies.

Retinal flat-mounts were blocked in blocking solution containing 3% donkey serum, 1% BSA, and 2% Triton™-X-100 in 1 x PBS at room temperature for 1 hour. Thereafter, flat-mounts were incubated in blocking solution with antibodies directed against ChAT (Millipore) and Brn3a (brain-specific homeobox/POU domain protein 3a, goat, 1:300, Santa Cruz Biotechnology, Dallas, TX, USA) at 4°C for 48 hours. Flat-mounts were washed in 1 x PBS and incubated with donkey anti-goat secondary antibodies (1:300, Alexa Fluor 488, Jackson ImmunoResearch) and TO-PRO™-3 iodide (1:400, Thermo Fisher Scientific, Invitrogen) in blocking solution without Triton™-X-100 for 2 hours at room temperature. Additionally, flat-mounts that were exposed only to secondary antibodies and TO-PRO™-3 iodide, without prior treatment with primary antibodies, were employed as negative controls.

For microscopic analyses, retinal cross-sections and flat-mounts were cover-slipped with ImmuMount (Thermo Scientific Shandon™). For retinal section analyses, images were taken from 2 retinal sections per slide. 2 peripheral and 2 central images per retina at a 400 x magnification were captured. To examine VGLUT1 staining, images (IPL: 100 x 240 pixel, OPL: 100 x 70 pixel), were cropped with Coral Paint Shop Pro X8 (Coral Corporation, Ottawa, Canada). A macro of the ImageJ software (National Institutes of Health, Bethesda, MD, USA) was used to perform masked evaluation of the staining signal area.^94^^,^^103^^,^^104^ Images were converted into 32-bit grey scale. The background was subtracted (rolling ball radius of 50 pixel). Then, the lower and upper threshold was determined manually by aligning the grayscale picture and the original one. The mean lower (IPL: 17.7 pixel, OPL: 28.8 pixel) as well as the upper threshold (IPL: 90.7 pixel, OPL: 86.4 pixel) were calculated and then used for the automated analysis. Means per retina were used for further statistical analysis. Cell counting was done for immunostainings of ChAT and GABA_A_ receptor α2. To analyze cell numbers in retinal flat-mounts, immunoreactive cells were counted in the peripheral and central retina (200 x magnification; 8 counting windows/retinal flat-mount per animal; 2 counting windows from each retinal quadrant). For the evaluation of the mean intensity of GABA_A_ receptor α2^+^ cells, we used the CellProfiler 4.2.1 software (Broad Institute, Cambridge, MA, USA).^105^ Images were converted from color to grayscale. GABA_A_ receptor α2^+^ cells were identified using the "IdentifyPrimaryObjects" module. We specified a "typical diameter of object in pixel units" ranging from a minimum of 10 to a maximum of 70 pixels. We utilized an "adaptive threshold strategy" with the "otsu thresholding method," employing "two-class thresholding" with lower and upper bounds on the threshold set at 0.2 and 1.0, respectively. Next, we incorporated the "measure object intensity" module to calculate the mean intensity of the identified cells. For each genotype, we recorded data from eight retinae. Within each retina, we captured two images in both the central and peripheral regions. Subsequently, we calculated the mean intensity of all measured cells within each retina, which was used for statistical analysis.

#### Western blot

Western blot analyses were conducted in accordance with a described protocol.^22^ For the analyses, P12 retinae of quadruple KO and WT mice were incubated in 80 μl of lysis buffer, consisted of 60 mM n-octyl-β-D-glucopyranoside, 50 mM sodium acetate, 50 mM tris chloride at pH 8.0, and a protease inhibitor cocktail (Sigma-Aldrich) on ice for a duration of 1 hour and triturated with a pipette tip. Following this, the samples were centrifuged at 14,000 × g at 4°C for 30 minutes. Then, the protein concentration in the resulting supernatant was determined using the bicinchoninic acid assay (BCA) Protein Assay Kit from Pierce (Thermo Fisher Scientific), conducted according to the manufacturer's guidelines. To each protein sample (10-20 μg per lane), 4 × SDS buffer was added, and the samples were denatured for 5 minutes at 95°C. Subsequently, the proteins were separated through SDS-PAGE utilizing 4-10% polyacrylamide gels, and then they were transferred onto a polyvinylidene difluoride (PVDF) membrane via the Western blotting technique for 1.5 hours. The Precision Plus Protein Standards Dual Color Standards (Bio-Rad Laboratories GmbH, München, Germany) was used for molecular weight estimation. Following the transfer, the PVDF membranes were subjected to blocking in blocking solution, comprising 5% w/v milk powder in Tris-buffered saline (TBS) with 0.05% Tween 20 (TBST) at room temperature for 1 hour. The following primary antibodies were diluted in blocking solution: anti-actin (mouse, 1:5,000, Sigma-Aldrich), anti-Bcan (mouse, 1:5,000, BD Biosciences), anti-Ncan (sheep, 1:200, R&D Systems), anti-Tnc (batch KAF14/1, rabbit, 1:10,000,^85^), and anti-Tnr (anti-restrictin, clone 23-14, mouse, 1:1,000,^86^) and the membranes were incubated at 4°C overnight. The membranes were subsequently washed in TBST and exposed to horseradish peroxidase (HRP)-conjugated secondary antibodies (anti-mouse HRP, anti-rabbit HRP, anti-sheep HRP) diluted in blocking solution (1:3,000 - 1:10,000) at room temperature for 1 hour. Any unbound antibodies were removed through washing with TBST, followed by a final wash with TBS. Thereafter, a mixture of ECL substrate solutions at a 1:1 ratio (Bio-Rad Laboratories GmbH) was applied to the membranes for 5 minutes. The immunoreactivity of the proteins was then recorded using a MicroChemi Chemiluminescence Reader (Biostep, Burkhardtsdorf, Germany).

#### Microscopy and image processing

For the documentation of the *in-situ* hybridization, images were acquired using the Axioplan2 microscope equipped with an AxioCam MRm digital camera and the AxioVision 4.5 software (Carl Zeiss, Oberkochen, Germany). Fluorescence specimens were examined by confocal laser-scanning microscopy (LSM 510 META, Carl Zeiss). Laser lines and emission filters were adjusted using the Zeiss Black ZEN software (Carl Zeiss). Images were exported and processed with Adobe Photoshop and Adobe Illustrator (Adobe, Dublin, Ireland), Coral Paint Shop Pro X8 (Coral Corporation), and ImageJ (National Institutes of Health).

### Quantification and statistical analysis

For IOP analyses, both eyes were measured and 10 readings of each eye were averaged. Data (N = 12) were compared by Student`s *t*-test (STATISTICA, V13.3, StatSoft, Hamburg, Germany) and are presented as mean ± standard error of the mean (SEM). ERG data (N = 8) were compared with STATISTICA using one-way analysis of variance (ANOVA) followed by Tukey`s post-hoc test and are shown as mean ± SEM. OMR data (N = 6) were compared with STATISTICA using one-way ANOVA followed by Tukey`s post-hoc test and are shown as mean ± SEM. MEA data of quadruple KO and WT mice (N = 10) were analyzed in MATLAB (The MathWorks) and are shown as mean ± SEM. NGS data of WT and quadruple KO retinae (N = 4) were bioinformatically analyzed as described in detail above. Heatmaps show the normalized expression (log2 scale) of significantly regulated genes (adjusted p-value < 0.05) annotated with GO terms for the groups of interest. Data of immunohistochemical analyses (N = 3-8) were presented as mean ± SEM and/or ± standard deviation (SD) and groups were analyzed with Matlab (MathWorks) or Statistica using Student`s *t*-test or one-way ANOVA followed by Tukey`s post-hoc test. Values of p < 0.05 were considered statistically significant.

## Supplemental information

Document S1. Figures S1–S9 and Tables S1, S3, and S4

Table S2. Most prominently down- and upregulated genes (adjusted p-value <0.05) in the retina of quadruple KO mice compared with WT mice as revealed by NGS, related to Figure 7
