# Neural extracellular matrix regulates visual sensory motor integration

**DOI:** 10.1016/j.isci.2026.115132

**Published:** 2026-02-26

**Authors:** Jacqueline Reinhard, Cornelius Mueller-Buehl, Susanne Wiemann, Lars Roll, Veronika Luft, Hamed Shabani, Daniel L. Rathbun, Lin Gan, Chao-Chung Kuo, Julia Franzen, Stephanie C. Joachim, Andreas Faissner

## Main text

(iScience *27*, 108846; February 16, 2024)

In Figure S1 of the originally published version of this article, panels K and K’ incorrectly displayed duplicate images of panels P and P’, which show *in situ* hybridizations for the gene *Tnr* instead of the intended *Tnc* sense and antisense images. The authors have provided corrected images for panels K and K’, accurately representing the *Tnc* hybridizations. This correction does not affect the main article text, data, or conclusions, and the updated image is shown below. The authors apologize for any confusion this error may have caused.

**Figure:**
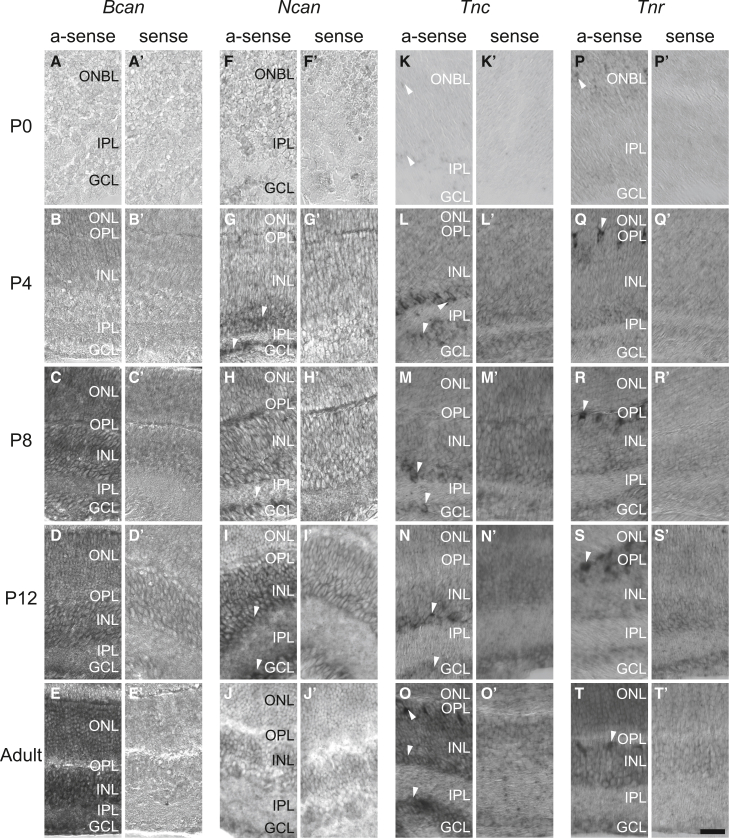
Figure S1. *In situ* hybridization revealing the spatiotemporal expression pattern of Bcan, Ncan, Tnc and Tnr in the postnatal and adult mouse retina, related to Figure 1

