# Supplementary material for: Neural extracellular matrix regulates visual sensory motor integration
Source: iScience. 2024 Jan 9;27(2):108846. doi: 10.1016/j.isci.2024.108846 (PMC10839651; doi:10.1016/j.isci.2024.108846)
Supplement: Document S1. Figures S1–S9 and Tables S1, S3, and S4 [file mmc1.pdf]

iScience, Volume *27*

Supplemental information

Neural extracellular matrix regulates visual sensory motor integration

Jacqueline Reinhard, Cornelius Mueller-Buehl, Susanne Wiemann, Lars Roll, Veronika Luft, Hamed Shabani, Daniel L. Rathbun, Lin Gan, Chao-Chung Kuo, Julia Franzen, Stephanie C. Joachim, and Andreas Faissner

Supplement

Suppl. figures and tables

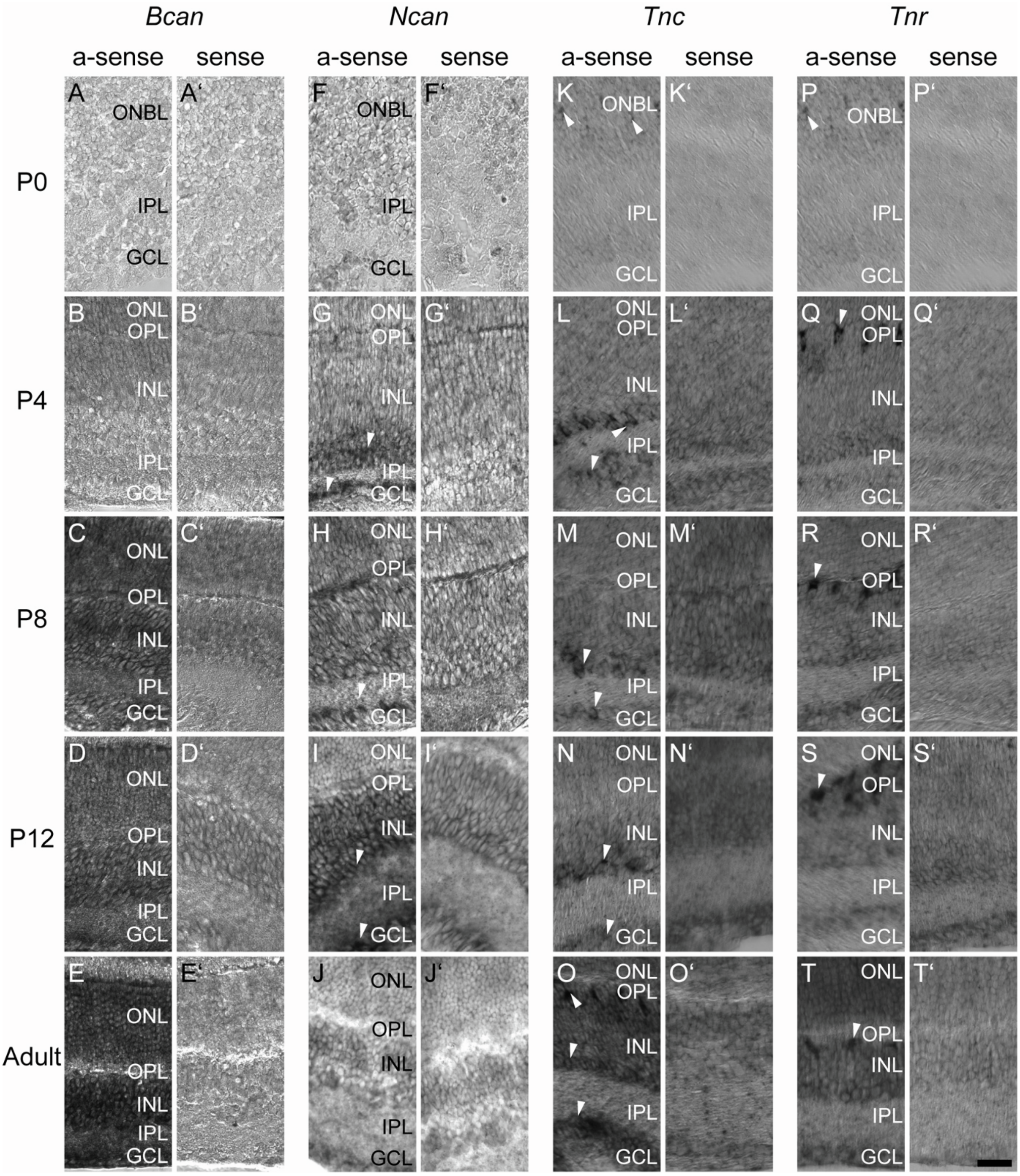

Figure S1. *In situ* hybridization revealing the spatiotemporal expression pattern of *Bcan*, *Ncan*, *Tnc*, and *Tnr* in the postnatal and adult mouse retina, related to Figure 1.

Differential mRNA expression pattern of *Bcan* (A-E, anti-sense; A`-E`, sense), *Ncan* (F-J, anti-sense; F`-J`, sense), *Tnc* (K-O, anti-sense; K`-O`, sense), and *Tnr* (P-T, anti-sense; P`-T`, sense) in the postnatal (P0, P4, P8 and P12) and adult retina. White

arrows mark prominent signals. Scale bar: 20 µm. *Bcan* = *brevican*, GCL = ganglion cell layer, INL = inner nuclear layer, IPL = inner plexiform layer, *Ncan* = *neurocan*, ONBL = outer neuroblastic layer, ONL = outer nuclear layer, OPL = outer plexiform layer, P = postnatal, *Tnc* = *tenascin-C*, *Tnr* = *tenascin-R*.

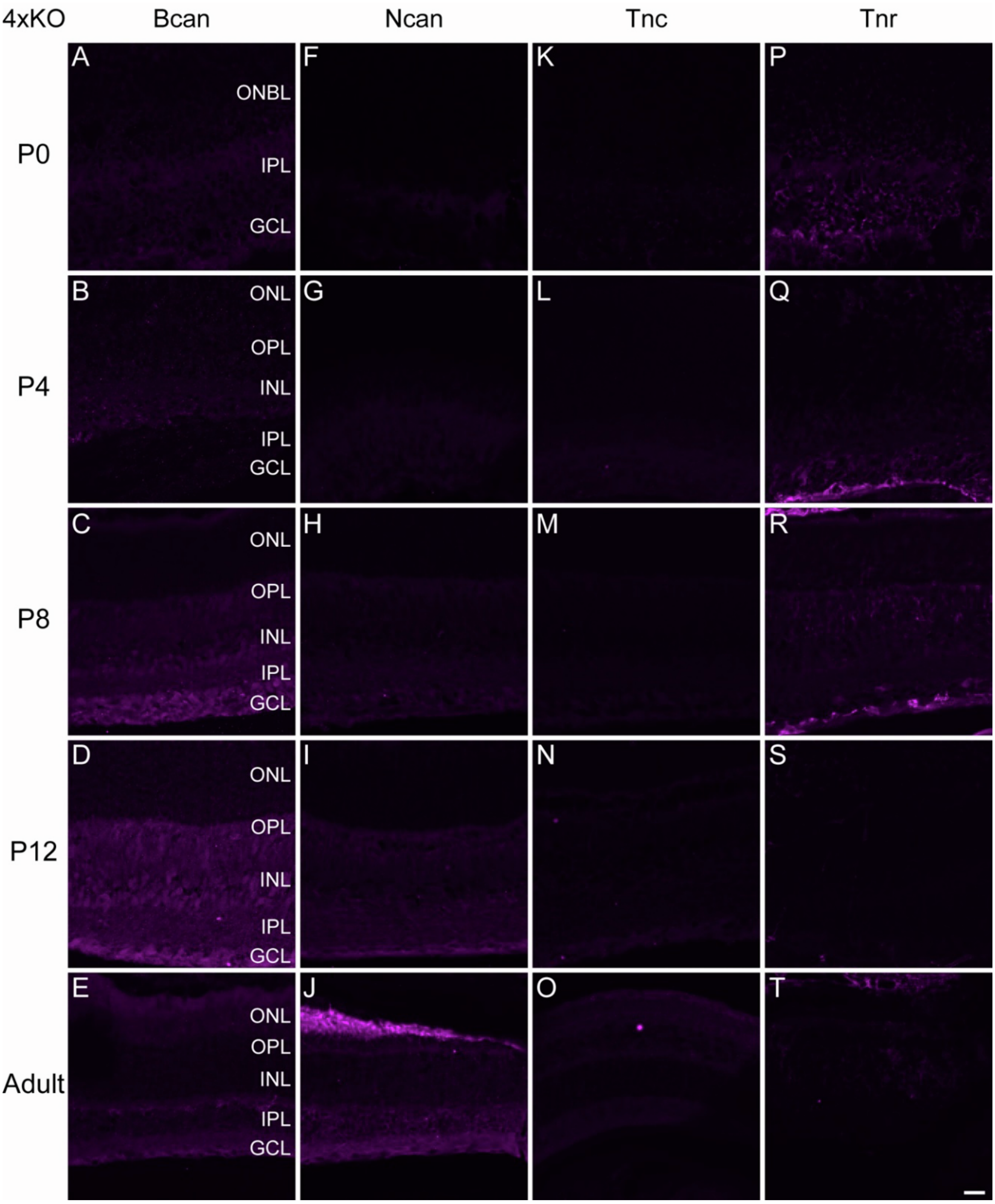

Figure S2. Control staining of Bcan, Ncan, Tnc and Tnr in the postnatal and adult quadruple KO retina, related to Figure 1.

Immunohistochemical staining of Bcan (A-E), Ncan (F-J), Tnc (K-O), and Tnr (P-T) in the postnatal (P0, P4, P8, and P12) and adult quadruple KO retina. The control staining

revealed only faint, if any, background staining in the retina lacking the four molecules. Scale bar: 20 µm. 4xKO = quadruple knockout, Bcan = brevican, GCL = ganglion cell layer, INL = inner nuclear layer, IPL = inner plexiform layer, Ncan = neurocan, ONBL = outer neuroblastic layer, ONL = outer nuclear layer, OPL = outer plexiform layer, P = postnatal, Tnc = tenascin-C, Tnr = tenascin-R.

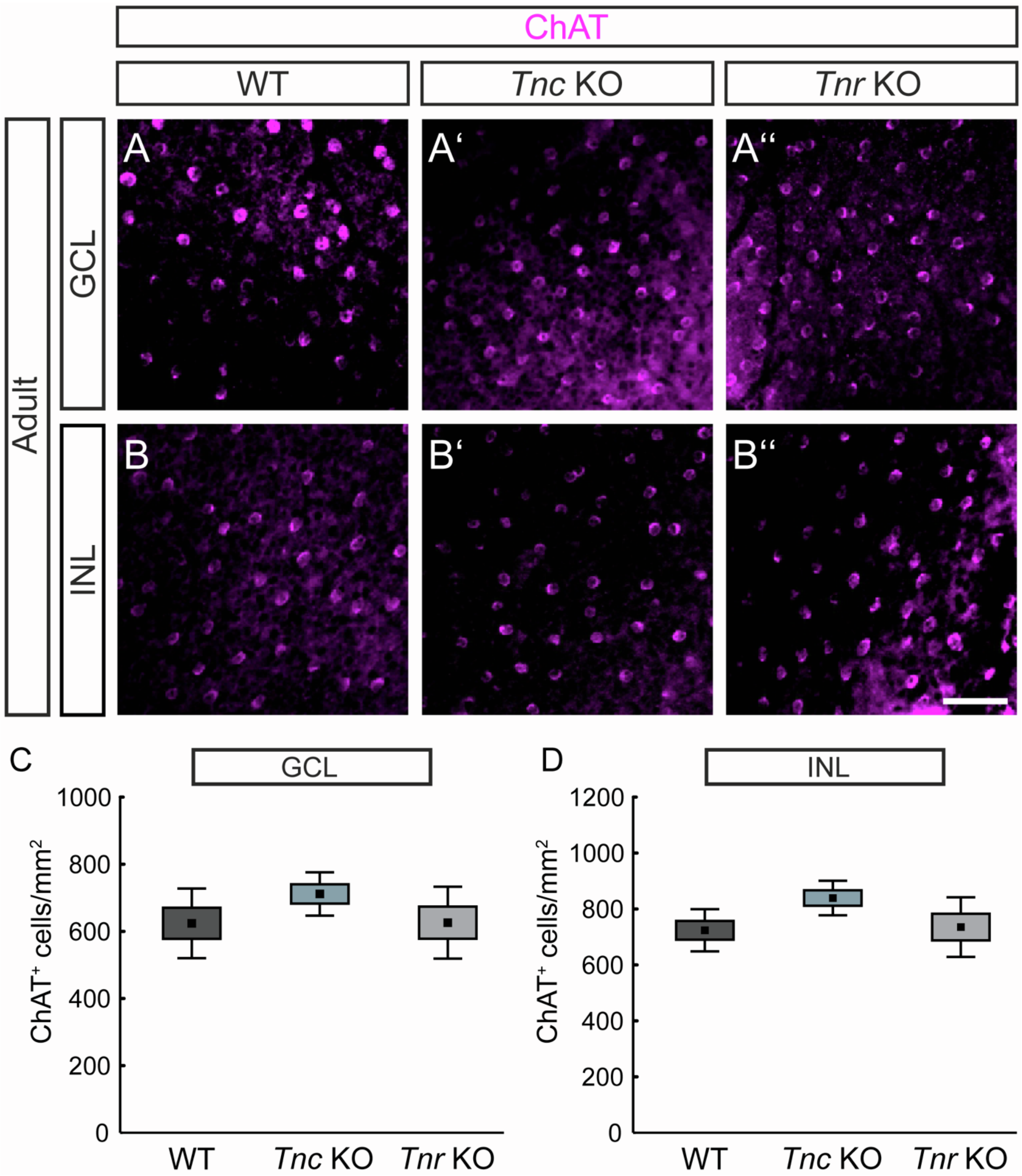

Figure S3. Staining of ChAT⁺ SACs in the *Tnc* KO and *Tnr* KO retina, related to Figure 4.

(A, B) Immunohistochemical staining of ChAT⁺ cells (magenta) in the adult *Tnc* and *Tnr* single KO retina. (C, D) Quantification revealed a similar number of ChAT⁺ SACs in the single KOs compared to WT mice. Scale bar: 50 µm. N = 5-7. GCL = ganglion cell layer, INL = inner nuclear layer, *Tnc* KO = *tenascin-C* knockout, *Tnr* KO= *tenascin-R* knockout, WT = wildtype. Data are shown as mean ± SEM ± SD.

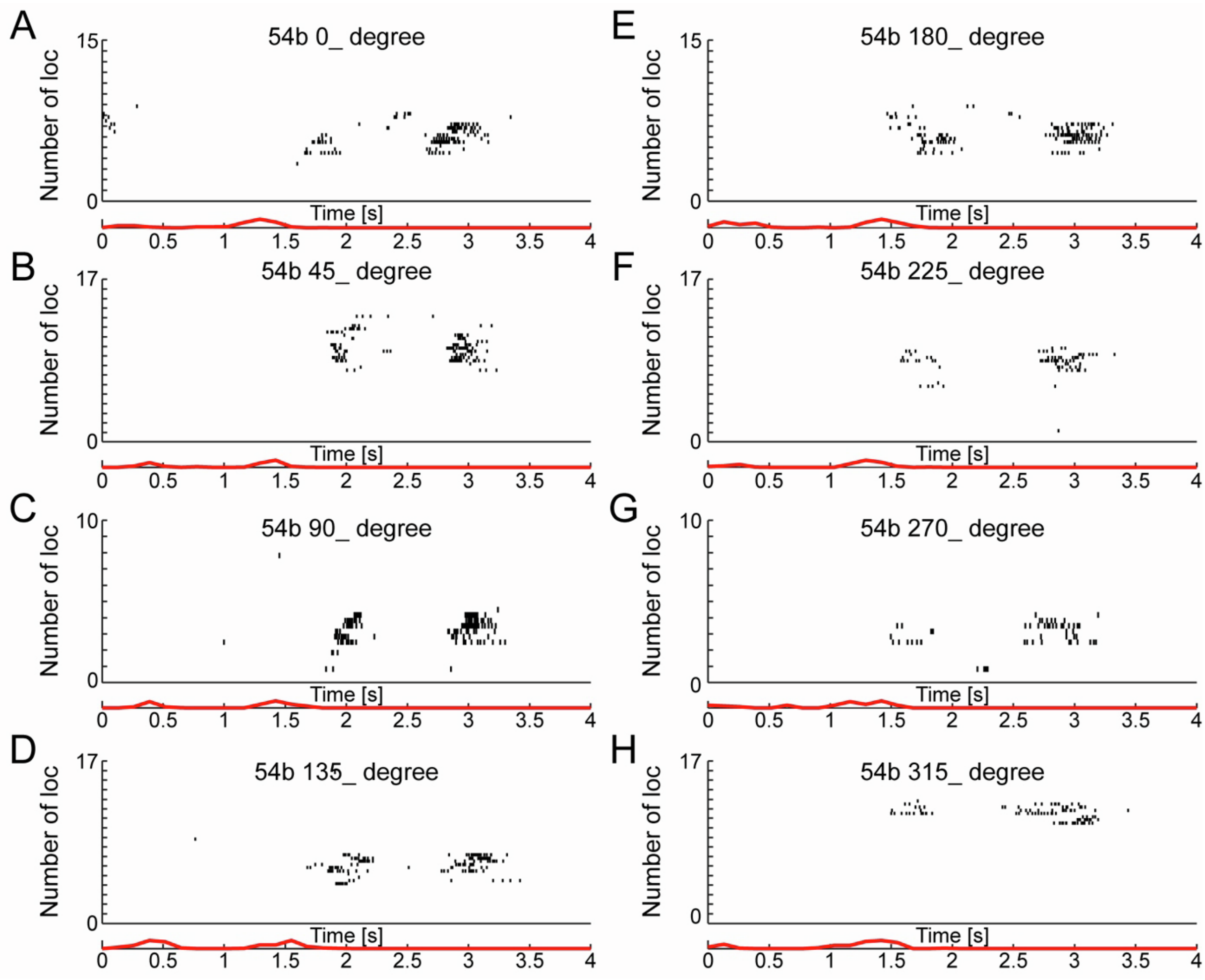

Figure S4. Responses of an individual DSGC, related to Figure 5.

(A-H) The diagrams depict the action potentials of an individual DSGC over time (s; 0°-315°). s = second.

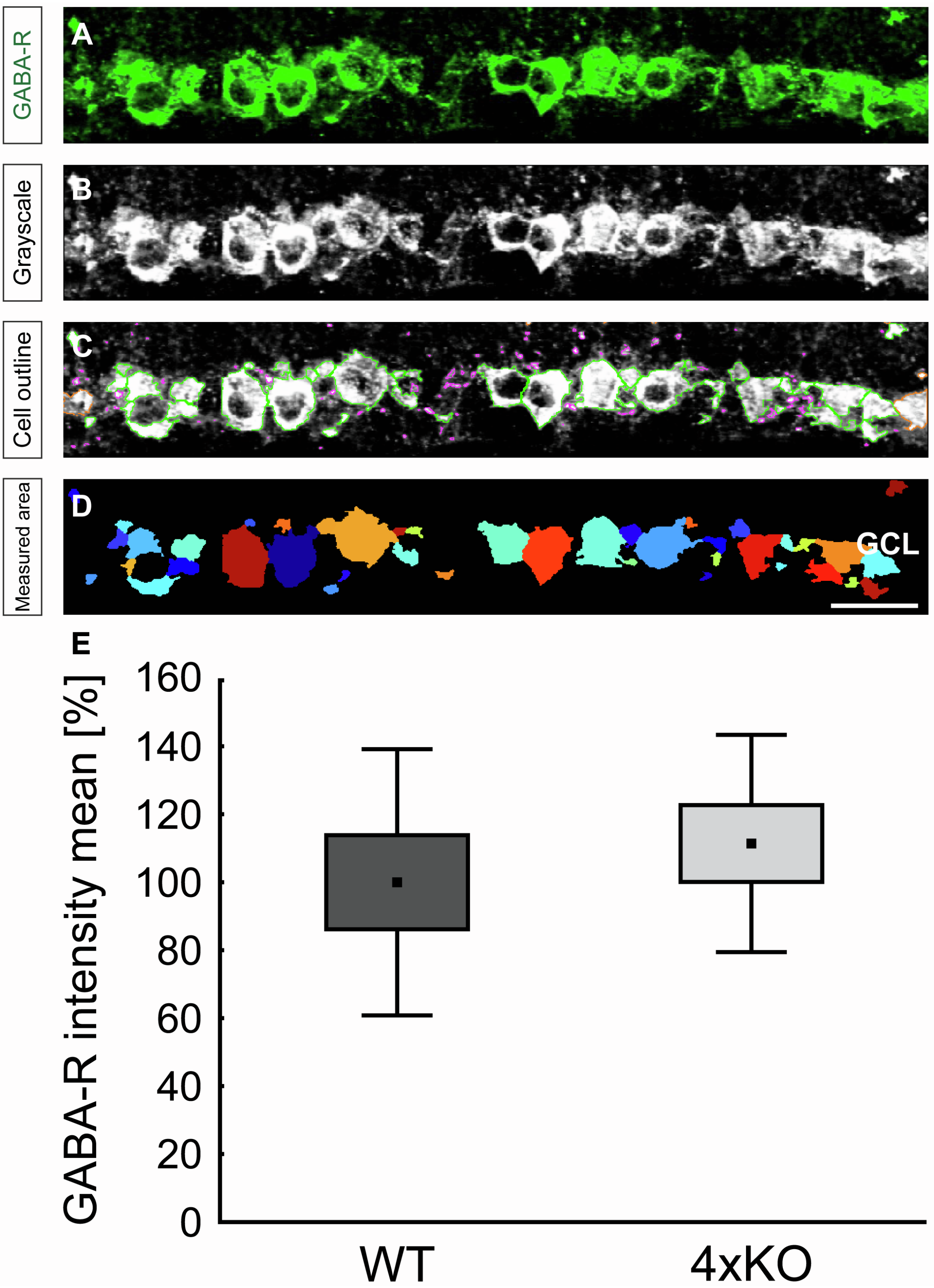

Figure S5. Mean intensity of $GABA_A$ receptor α2$^+$ cells, related to Figure 6.
(A-D) Exemplary staining and processing of GABA-R$^+$ cells in the adult retina. (E) Quantification revealed a similar mean GABA-R signal intensity in 4xKO compared to WT mice. Scale bars = 25 µm. N = 5. 4xKO = quadruple knockout, GABA-R = γ-aminobutyric acid A receptor α2, GCL = ganglion cell layer, WT = wildtype. Data are shown as mean ± SEM ± SD. WT were set to 100%.

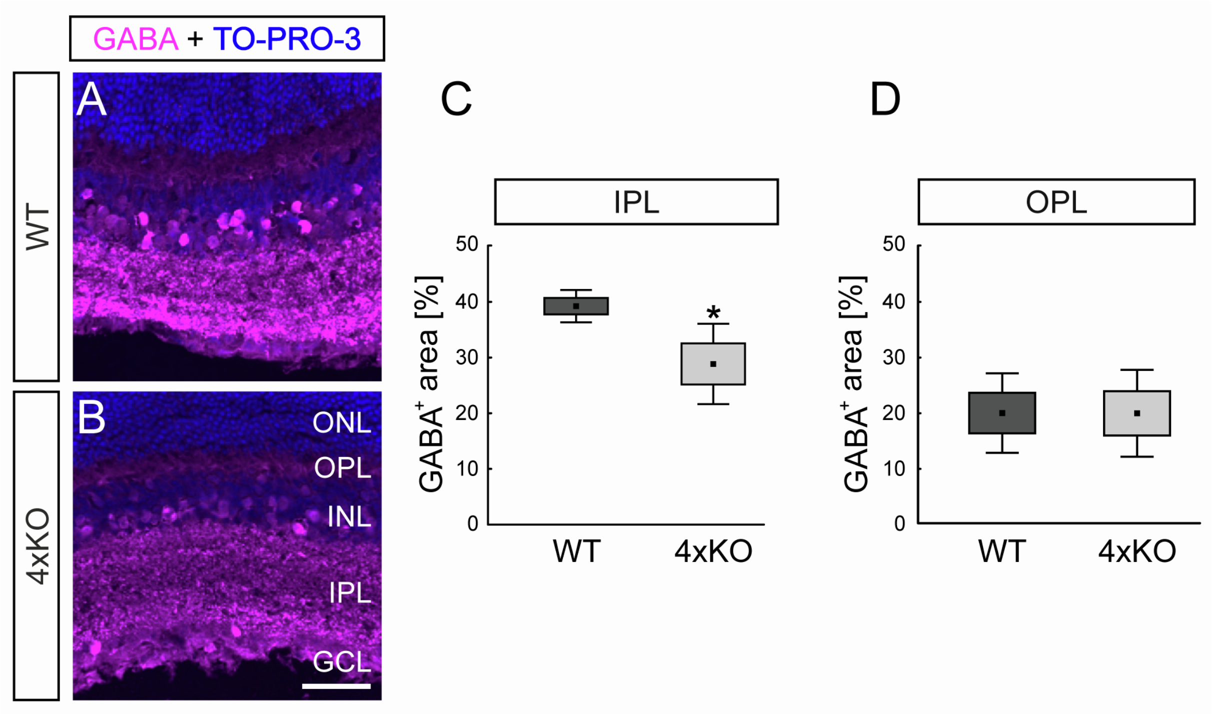

Figure S6. GABA staining in the WT and quadruple KO retina, related to Figure 6.

(A, B) Exemplary images of retinal sections of WT and quadruple KO mice, which were stained with anti-GABA (magenta). Cell nuclei were stained with TO-PRO-3 (blue). (C, D) Quantification revealed a significantly reduced GABA staining area in the IPL, while a comparable GABA staining was noted in the OPL. Scale bar = 50 µm. N = 5. * = $p < 0.05$. 4xKO = quadruple knockout, GABA = γ-aminobutyric acid, GCL = ganglion cell layer, INL = inner nuclear layer, IPL = inner plexiform layer, ONL = outer nuclear layer, OPL = outer plexiform layer, WT = wildtype. Data are shown as mean ± SEM ± SD.

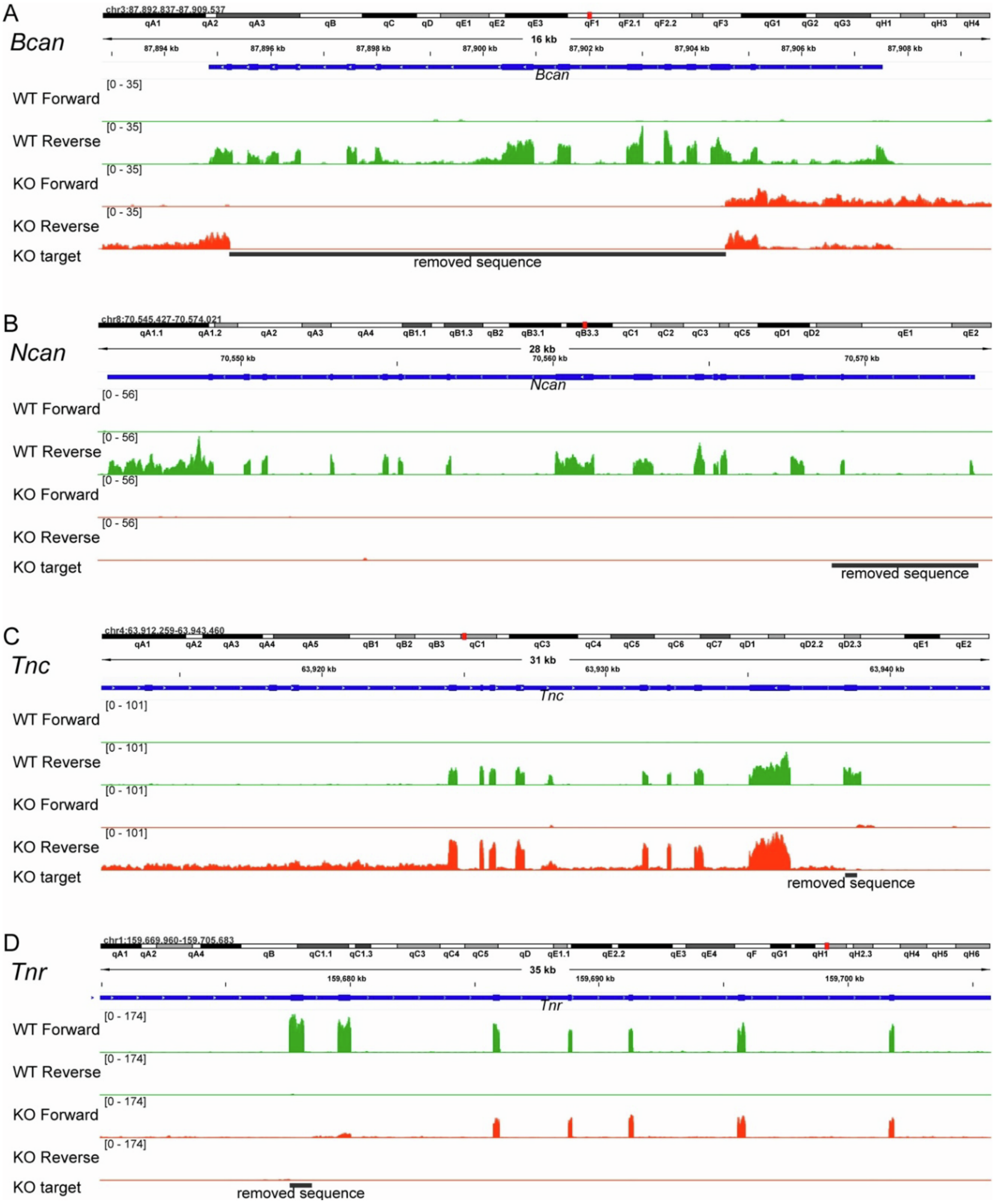

Figure S7. Results of the Integrative Genomics Viewer (IGV) for the *Bcan*, *Ncan*, *Tnc*, and *Tnr* alleles, related to STAR Methods.

(A) The *Bcan* WT sequence is lost between position 87,895,223 and 87,904,572, according to GRCm39 (NC_000069.7). (B) The *Ncan* WT sequence is lost between position 70,568,941 and 70,573,644, according to GRCm39 (NC_000074.7). (C) The *Tnc* WT sequence is lost between position 63,938,412 and 63,938,833, according to GRCm39 (NC_000070.7). (D) The *Tnr* WT sequence is lost between position 159,677,592 and 159,678,477, according to GRCm39 (NC_000067.7). (A-D) The WT is shown in green, while the KO is shown in red. KO = knockout, WT = wildtype.

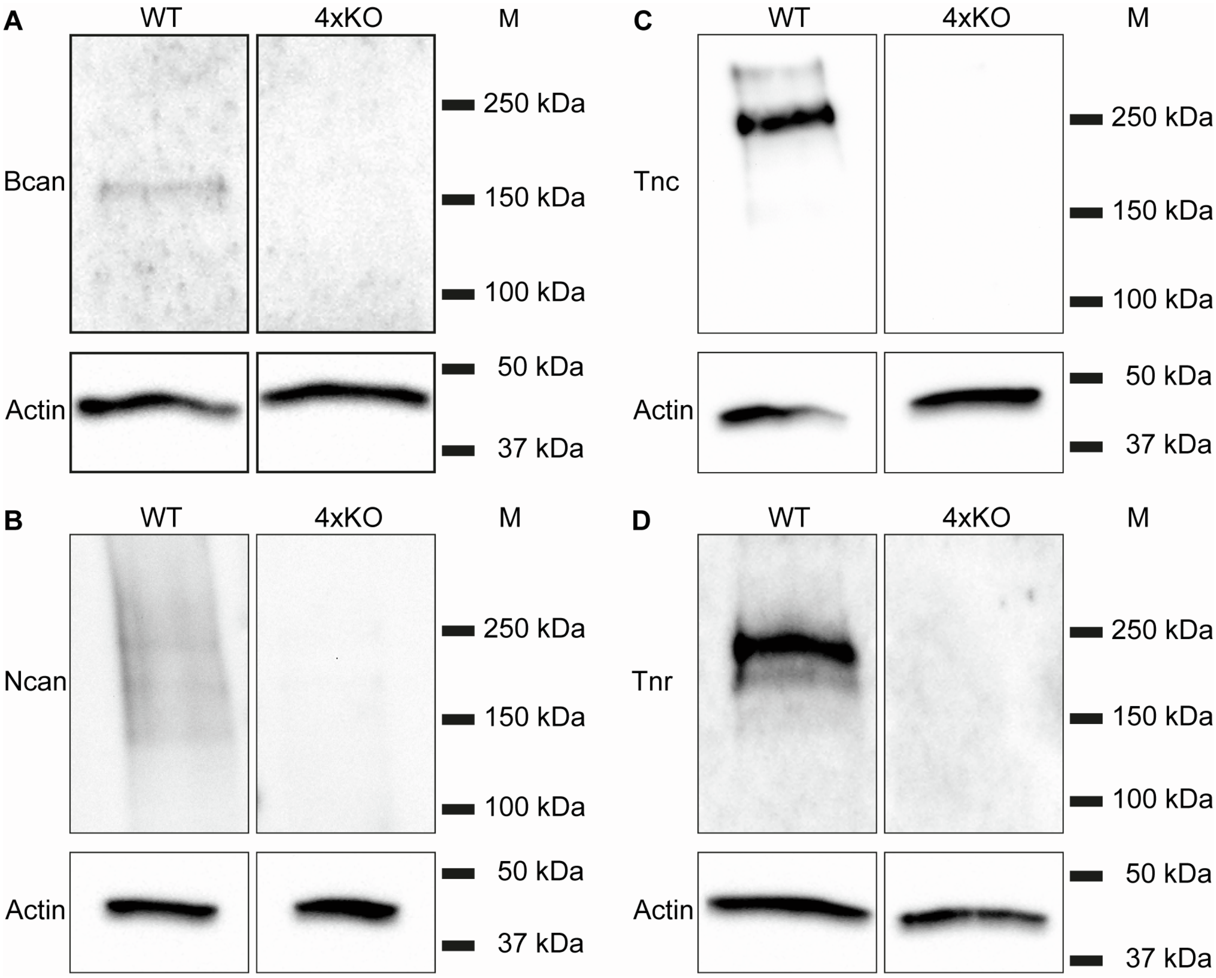

Figure S8. Western blot analyses revealed the absence of the Bcan, Ncan, Tnc, and Tnr proteins in the 4xKO retina, related to STAR Methods.

(A) In the WT retina, the anti-Bcan antibody detected the Bcan protein at ~140 kDa. (B) The anti-Ncan antibody detected Ncan at ~200 kDa. (C) The anti-Tnc antibody detected two bands at ~250 and >250 kDa. (D) The anti-Tnr antibody detected two bands at ~160 and ~180 kDa. Proteins were purified from P12 WT and 4xKO retinae. Bcan, Ncan, Tnc, and Tnr proteins were not specifically observed in the 4xKO condition, showing the loss of the four proteins in the KO as well as the specificity of the antibodies used. Protein loading of retinal lysates was verified by the detection of the protein actin at ~37 kDa. For molecular weight estimation, marker lanes were digitally transferred to the images. 4xKO = quadruple knockout, Bcan = brevican, kDa = kilodalton, M = marker, Ncan = neurocan, Tnc = tenascin-C, Tnr = tenascin-R, WT = wildtype.

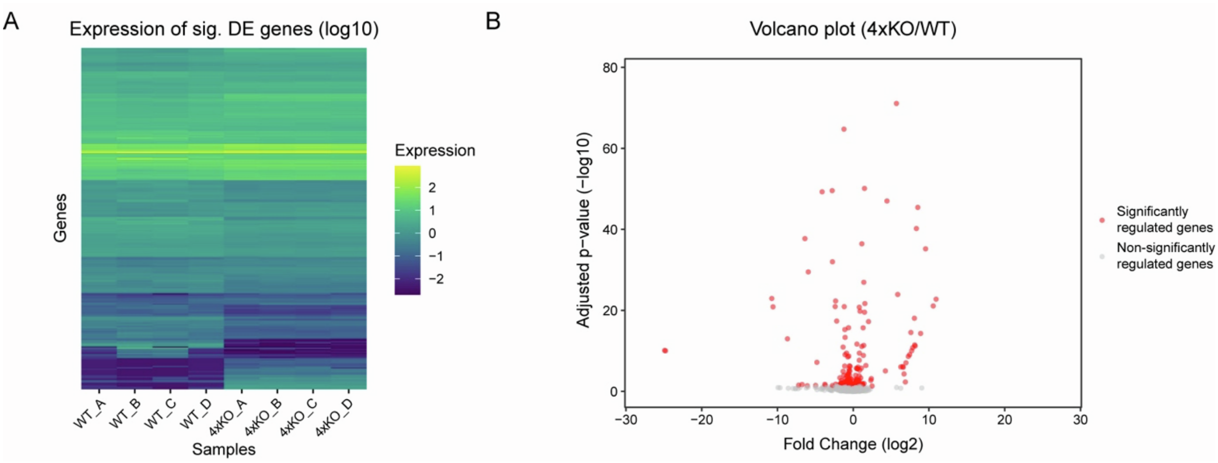

Figure S9. NGS analyses identified 263 genes significantly altered in the quadruple KO compared to the WT retina, related to Figure 7.

(A) The heatmap illustrates the significant differentially 263 expressed genes (log10) in the WT and KO samples. N = 4. The expression levels of these genes are indicated by the color range from dark blue to yellow. 4xKO = quadruple knockout; WT = wildtype. (B) Additionally, a volcano plot shows significantly up- and downregulated genes (red dots) as well as non-significantly regulated genes (grey) according to their adjusted p-value (-log10) and fold change (log2). 4xKO = quadruple knockout, WT = wildtype.

Table S1. A- and b-wave amplitudes recorded from adult quadruple KO and WT mice *via* ERG analyses, related to Figure 2. Light flash intensity, amplitude mean, SEM, genotypes and the p-value are indicated. P-values < 0.05 are shown in bold. 4xKO = quadruple knockout, cd * s/m^2 = candela x seconds per meter square, WT = wildtype.

**Light flash intensity [cd * s/m²]**	**0.1**		**0.3**		**1**		**3**		**10**		**25**	
**Amplitude [µV]**	**Mean**	**SEM**	**Mean**	**SEM**	**Mean**	**SEM**	**Mean**	**SEM**	**Mean**	**SEM**	**Mean**	**SEM**
**A-wave**												
WT	75.87	6.41	133.04	7.00	155.64	8.00	162.14	10.25	186.08	10.25	202.57	9.75
4xKO	59.36	3.88	107.09	5.82	129.61	6.78	144.28	5.47	156.36	8.73	184.60	11.03
P-value	**0.035**		**0.007**		**0.018**		**0.035**		**0.035**		0.232	
**B-wave**												
WT	483.34	19.64	552.33	26.26	560.28	25.80	517.33	21.68	618.24	29.80	628.26	29.11
4xKO	341.19	17.95	404.38	17.50	425.27	19.20	408.29	18.03	464.41	19.81	487.53	16.91
P-value	**< 0.001**		**< 0.001**		**< 0.001**		**< 0.001**		**< 0.001**		**< 0.001**	
**Implicit time [ms]**	**Mean**	**SEM**	**Mean**	**SEM**	**Mean**	**SEM**	**Mean**	**SEM**	**Mean**	**SEM**	**Mean**	**SEM**
**A-wave**												
WT	24.15	1.14	23.13	0.33	20.60	0.29	14.59	0.35	13.51	0.66	10.71	0.13
4xKO	23.63	0.51	21.98	0.45	19.16	0.38	13.34	0.21	12.84	0.48	10.68	0.18
P-value	0.678		0.051		**0.005**		**0.004**		0.418		0.866	
**B-wave**												
WT	64.80	1.39	62.91	1.35	64.31	1.51	69.62	1.58	64.79	1.30	63.53	2.06
4xKO	62.63	1.16	62.15	1.53	62.91	1.18	71.11	1.64	62.99	1.32	62.99	1.70
P-value	0.238		0.714		0.469		0.516		0.341		0.840	

Table S3. Primers for genotyping by PCR, related to STAR Methods.

The primer sequence, amplicon size and reference are listed. bp = base pairs, For = forward, Rev = reverse, neo = neomycin.

Primer	Sequence 5`→ 3`	Amplicon Size (bp)	Reference
3`neo*	GATTCGCAGCGCATCGCCTT	WT: ** & *** 222<br>KO: * & *** 636	This work
Bcan ForWT**	TATTAAGGAGGAGCGCCGTG		
Bcan Rev***	TCACCCCCTATCATGGGGAA		
Ncan ForWT	TCTTGGGGATGCCACGATTC	WT: 331	This work
Ncan RevWT	GGGAAACTCCACTGCTGGTTA		
Ncan ForKO	GAATCCCCACTCTGCCCTTT	KO: 1,115	
Ncan RevKO	ACCTGAGTCAGAGGTAGGGG		
Tnc For*	CTGCCAGGCATCTTTCTAGC	WT: * & *** 435<br>KO: ** & *** 342	[1]
Tnc neo For**	CTGCTCTTTACTGAAGGCTC		
Tnc Rev***	TTCTGCAGGTTGGAGGCAAC		
Tnr For*	AACTCCATGCTGGCTACCAC	WT: * & *** 420<br>KO: * & ** 429	[2]
Tnr neo Rev**	ACCGCTTCCTCGTGCTT		
Tnr Rev***	TTTTGGGGAGGTTGATCTTG		

Table S4. Primers for RT-PCR and the generation of *in situ* riboprobes, related to STAR Methods and Figure S1.

The primer sequence, amplicon size, GenBank accession number, and reference are listed. bp = base pairs, ISH = *in situ* hybridization.

Primer	Sequence 5`→ 3`	Amplicon size (bp)	GenBank accession number; reference
*Bcan*_ISH_forward	TCAAGTGGACCTTCCTGTCC	1,093	NM_007529; this work
*Bcan*_ISH_reverse	GACTCGGTAGGTGGTGCAAT		
*Ncan*_ISH_forward	TGCCACGCTCTACACTTGTC	1,062	NM_007789; this work
*Ncan*_ISH_reverse	GGCTGCATAAGCAGTCATCA		
*Tnc*_ISH_foward	CCATGGGTTCTCCGAAGGAAA	1,211	NM_011607; [2]
*Tnc*_ISH_reverse	GGATACAGTGGAACAGCAGGT GACGTCACC		
*Tnr*_ISH_foward	GATAGCCCCATGGAT	1,215	NM_022312; [2]
*Tnr*_ISH_reverse	GAATTTCAAGG		

SUPPLEMENTAL REFERENCES

[1] Talts, J.F., Wirl, G., Dictor, M., Muller, W.J., Fassler, R. (1999). Tenascin-C modulates tumor stroma and monocyte/macrophage recruitment but not tumor growth or metastasis in a mouse strain with spontaneous mammary cancer. J. Cell Sci. *112*, 1855-1864.

[2] Czopka, T., Von Holst, A., Schmidt, G., Ffrench-Constant, C., and Faissner, A. (2009). Tenascin C and tenascin R similarly prevent the formation of myelin membranes in a RhoA-dependent manner, but antagonistically regulate the expression of myelin basic protein via a separate pathway. Glia *57*, 1790-1801.
